# Leveraging digital solutions to address gender inequalities in EU agriculture: a CODECS framework application

**DOI:** 10.12688/openreseurope.21445.1

**Published:** 2025-10-17

**Authors:** Barbra Comfort Akello, Gianluca Brunori

**Affiliations:** 1 Department of Agricultural, Food and Agro-Environmental Sciences, University of Pisa, Pisa, 56124, Italy

**Keywords:** Gender inequality, Agriculture, Digitalisation, CODECS, EU policies

## Abstract

**Background:**

Despite longstanding EU commitments to gender equality, structural disparities continue to restrict women’s participation and leadership in agriculture and rural development. Digitalisation is increasingly promoted as a catalyst for addressing these inequalities. However, there is a limited holistic understanding of the conditions under which digital innovations can foster inclusive and sustainable outcomes. A key barrier is the absence of integrative frameworks that can systematically categorise the multifaceted evidence emerging from digital transitions. This limitation hampers both the comparability of findings and their practical relevance for policymaking. To address this, the study employs the CODECS conceptual framework to evaluate how digitalisation intersects with gender inequality in EU agriculture.

**Methods:**

Drawing on peer-reviewed literature, policy analyses, technical reports and empirical data from CODECS Living Labs, the study explores dynamics across three levels: business process digitisation, ecosystem-wide digitalisation, and broader socio-ecological systems transformations.

**Results:**

The analysis reveals key persistent gaps, including gender-biased technology design, socio-cultural norms, low digital confidence, financial exclusion, and data disempowerment. However, it also identifies opportunities, particularly when digital tools are designed with context-awareness and co-created through participatory approaches. Living Labs demonstrate how innovations such as accessible advisory services, digital marketplaces, and moderated learning platforms can empower rural women by increasing their visibility, agency, and access to resources.

**Conclusions:**

Digital technologies alone cannot overcome entrenched inequalities. Promoting equity requires complementary reforms in governance, institutional structures, and data accountability. The CODECS framework provides a structured lens for understanding and addressing gendered dynamics in agricultural digitalisation, offering practical insights for inclusive innovation and evidence-based policymaking in Europe.

## Plain Language Summary

In rural Europe, women in farming face significant challenges. Despite carrying out a large portion of the agricultural work, they often have less access to land, income, education, and decision-making power than men. This inequality makes it more difficult for women to operate successful farms, earn a fair living, or have a strong voice in their communities. This study looks at whether digital technologies such as soil sensors, crop-monitoring drones, and digital marketplaces could help reduce these gender gaps.

To investigate this, the authors use the multi-level CODECS framework that looks at the interactions between day-to-day farm work, the surrounding digital services and markets, and broader national policies and social norms. This approach helps reveal how gender inequalities appear at each of these levels and helps identify ways to address them. The research highlights real-world examples from across Europe where digital platforms have successfully helped women farmers by increasing their visibility, providing access to new markets, saving them time, and building supportive networks. However, it also cautions that if digital tools are not designed with women’s needs in mind, they could unintentionally make existing inequalities worse. Finally, the study offers practical policy suggestions, such as using co-design strategies that include women, improving data collection on women in agriculture, and ensuring that online spaces are both safe and accessible.

## Introduction

Agriculture in the European Union (EU) remains characterised by deep-rooted gender inequalities, despite decades of political efforts to achieve equality ^1^. Women continue to face significant barriers in accessing land, finance, education, technology, as well as meaningful decision-making power, leaving them at a disadvantage compared to men in almost all areas of agriculture and rural life ^2^. Although gender equality is frequently cited in rural development objectives, many initiatives fail to address the underlying socio-cultural structures that reinforce this marginalisation ^3^. As Shortall and Marangudakis ^4^ identify, a fundamental structural imbalance of influence underpins the ineffectiveness of these measures. Dominant agricultural lobbying groups wield substantial influence over EU policy priorities, while gender-focused advocacy remains comparatively under-resourced and lacks robust legal and institutional backing. As a result, gender concerns are systematically sidelined in EU’s agricultural policy framework.

The outcome of this neglect is adverse for women in rural areas, contributing to lower productivity, fewer entrepreneurial opportunities, limited economic independence, and diminished quality of life ^1, 4, 5^. This marginalisation is further evidenced by the reality that although women make up 35% of the agricultural labour force in the EU and contribute 31% of total working hours to the economy, their work continues to be undervalued, and their specific needs often remain unaddressed in policy and practice ^6^. This persistent gender gap contrasts with the EU’s long-standing commitment to gender equality, enshrined in its treaties and reinforced by milestones such as the 1957 Treaty of Rome, the 1996 communication on gender mainstreaming, and the Gender Equality Strategy 2020–2025 ^7^. 

Digitalisation in agriculture is widely promoted in policy and industry circles as a strategic opportunity to redress the structural imbalances ^8^. This techno-optimist discourse positions the so-called fourth agricultural revolution, driven by innovations such as artificial intelligence (AI), blockchain, and the Internet of Things (IoT), as a potent force for levelling the playing field ^9– 11^. The potential pathways for this transformation are diverse. When thoughtfully designed and embedded in user-oriented applications, digital technologies can empower marginalised groups, particularly women, by mitigating gender barriers. For instance, mobile-based advisory services can bypass male-dominated extension networks, delivering tailored information directly to female farmers ^12^. Digital financial platforms (FinTech) offer mechanisms to enhance financial inclusion thereby strengthening women's economic agency ^13^. Furthermore, IoT and automation technologies hold the promise of reducing the time and labour intensity of tasks predominantly shouldered by women, freeing up time for education, entrepreneurial activities, or civic participation ^9^.

Research on digitalisation in EU agriculture has expanded considerably, addressing topics ranging from technological innovation and precision agriculture to environmental sustainability and rural integration ^2^. However, existing studies vary widely in terms of conceptual focus, methodology, and results, often providing fragmentary findings. Some emphasise the economic and productive benefits of digital technologies ^11, 14^, while others point to their potential to reduce rural poverty and eliminate gender inequalities ^4, 5^. Nevertheless, there is inadequate integrated analysis of the social, economic, and institutional conditions under which digitalisation can promote equitable and sustainable change. This gap is primarily due to the limited use of comprehensive frameworks capable of structuring and categorising the complex and multidimensional evidence for digitalisation ^9^. This lack of a synthesising framework complicates the interpretation of results and limits their usefulness for evidence-based decision-making.

Moreover, the current efforts to digitalise agriculture are often driven by top-down, industrial visions that overlook existing social inequalities ^15, 16^. These approaches render the specific needs, capabilities, and constraints of vulnerable groups like women invisible. This oversight creates a critical knowledge gap, as there remains little empirical evidence on how digital technologies can overcome these barriers to genuinely reduce gender inequalities ^17^. Without targeted inclusion strategies that address this complex reality, digitalisation risks reinforcing the very disparities it aims to overcome. This underscores the necessity to understand digitalisation not merely as a technological process, but as a profound social, and institutional transformation that requires gender-sensitive design, governance, and evaluation ^8, 16^.

To address these interconnected gaps, we draw on the framework developed by the EU Horizon project, Maximising the CO-benefits of agricultural Digitalisation through conducive digital ECoSystems (CODECS). The CODECS project provides a valuable integrated framework designed to structure and analyse the complex evidence surrounding digitalisation ^18^. By integrating social, economic, environmental, institutional, and technological perspectives, it enables a systematic synthesis of findings, bridging disciplinary boundaries and linking local experiences to EU policy objectives. In particular, we seek to answer the following questions:

1. What systemic gender-related barriers affect women’s participation and leadership in EU agriculture and rural areas?
2. How can digital technologies and ecosystems be designed to address these barriers in an inclusive and contextualised way?

Our contribution differs from previous studies in two ways. First, it synthesises evidence from technological, social, and institutional domains, moving beyond a narrow assessment of productivity gains to consider structural and gender inequalities. Second, it critically examines the mechanisms through which digitalisation can either mitigate or exacerbate gender inequalities, and identifies the necessary conditions for inclusive digital ecosystems. In doing so, the findings contribute to supporting the EU's ambitious Digital Decade goals, which seek to ensure that the benefits of digitalisation are accessible to all, especially groups at risk of exclusion, such as women.

## The CODECS framework

To analyse the transformative potential of digital solutions in addressing gender inequalities in EU agriculture and rural areas, this study employs the conceptual framework of CODECS as a theoretical basis. The framework developed within the CODECS project by Soma *et al*. ^18^ provides a multi-level, systems-based approach to understanding the dynamics, costs and benefits of digitalisation of agriculture, particularly in terms of social equity, environmental sustainability and institutional governance. The choice of the CODECS framework is motivated by the fact that it explicitly recognises digitalisation as a socially embedded and multi-scalar process rather than a mere technological upgrade. Digital technologies do not operate in isolation, but their development, uptake and impact are determined by complex interactions between technical capabilities and broader socio-political, economic and environmental forces. This realisation is particularly important when considering gender, as exclusion is not simply a question of access to tools, but a function of deep-rooted asymmetries in power, voice and opportunity.

The framework is organised into three interconnected levels, each representing different stages and scales of digital transformation:

Level 1: *Digitisation of business processes* focuses on individual farms and their use of digital tools for specific operational processes. At this stage, digital transformation is already influenced by external factors and leads to context-specific social, economic and environmental outcomes. Understanding how digitalisation operates at this level is essential for recognising the initial barriers that women face when engaging with digital farming. 

Level 2: *Digitalisation of ecosystems* goes beyond individual actors and extends to broader networks in the agricultural and food sector. It involves stronger networking of stakeholders, greater geographical reach and more complex interactions. This level leads to greater variation in sustainability impacts, characterised by feedback loops, relationships between actors and governance structures. This enhances the understanding of how gendered dynamics shape and are shaped by digital ecosystem configurations.

Level 3: *Digital transformation of socio-ecological systems (SES)* involves the comprehensive digitalisation of entire socio-ecological systems. Here, digital technologies influence natural resources, institutional arrangements and societal structures, including persistent inequalities. The SES level considers cumulative and cross-scale impacts, recognising that digitalisation has the dual potential to either reinforce existing social hierarchies, such as the gendered division of labour and access to resources, or to act as a driver of structural change toward greater equity.

A key strength of the CODECS framework lies in its context sensitivity. It situates digitalisation within an evolving socio-technical environment shaped by both external factors (e.g., political shifts, environmental changes, technological advancements) and internal dynamics (e.g., the capabilities, motivations, and opportunities of actors). In the context of gendered research, this sensitivity allows for a nuanced understanding of how women’s digital engagement is conditioned by local cultural norms, institutional support structures, and intersecting vulnerabilities. This perspective offers a theoretically coherent and empirically grounded basis for assessing whether, how, and under what conditions digital technologies can act as effective levers for promoting gender equality in EU agriculture and rural areas.

## Methodological approach

The analysis presented in this paper is grounded in the multi-scalar conceptual structure of the CODECS framework. We integrated insights from peer-reviewed literature, institutional reports, and policy documents, further enriched by empirical cases from CODECS Living Labs. These cases were developed through iterative, participatory processes, enabling a nuanced and in-depth analysis of digitisation of business processes, their resultant digital ecosystem configurations, and their embeddedness within broader socio-ecological systems.

This methodological orientation ensures that gender-related dynamics are not examined in isolation. Instead, they are understood as complex phenomena emergent from, and constitutive of the broader systemic interactions within digitalising agri-food systems.

## Barriers to gender inclusivity in agriculture and rural areas

Empirical evidence reveals a growing scholarly and institutional recognition of the critical role that gender dynamics play in shaping agricultural outcomes and rural livelihoods ^7^. However, as noted by Ball ^19^ and Fanelli ^6^, a pronounced imbalance remains. While the literature is comparatively robust for developing countries, studies focusing on women in agriculture in developed contexts remain relatively scarce. This disparity could be attributed to the fact that in many developing countries, women constitute a substantial proportion of the agricultural labour force and often face more acute socio-economic, legal, and institutional barriers than their counterparts in developed nations ^13^. Nevertheless, recent findings suggest that gendered challenges are not confined to the Global South. For instance, Streimikis and Kyriakopoulos ^1^, observe that the number of women entering farming roles in developed nations particularly across Europe has been rising steadily over the past decade. Understanding the specific challenges they face is crucial for designing inclusive and sustainable policy initiatives. To explore these barriers in greater depth, we present an overview of key gender disparities in agriculture and rural life across European contexts as shown in Table 1. These are organised using the three-tiered structure of the CODECS conceptual framework providing a structured perspective on these complex and evolving issues.

**Table 1.** Key gender barriers in agriculture and rural areas across Europe mapped to the CODECS framework's digitalisation levels.

Reference	Main area of contribution	Scope of the study
**Level 1: Digitisation of business processes**		
*Barriers related to socio-cultural norms*		
20	Female participation in farming and reasons for low rate of female farm ownership	Ireland
21	Depth of changes to contested gender identities in a (still) male dominated agricultural sector	Greece
22	Gender inequality in agriculture	Lithuania
23	Motivations and barriers for girls and women in STEM and ICT domains	Moldova
24	Persistence of gendered occupational closure	Scotland
*Time and labour burden*		
6	Identification of differences between male and female operated farms	EU
25	Tracking progress against long-lasting challenges regarding gender equality	EU
26	The European Parliament’s work on women in rural areas	EU
27	Examining the mismatch between European legislation on employment and women's employment position in agriculture	EU
28	Measuring gender gaps in both paid and unpaid work	EU
29	Understanding the role of labour market characteristics	EU
*Limited access to information*		
30	Analysis of the gender gap in entrepreneurial income within Italy’s farm sector	Italy
*Low digital literacy and confidence*		
31	Gender equality statistics in agricultural and digital sectors	EU
32	Analysing gaps in education levels between rural and urban areas	EU
33	Rationales and recommendations for gender equal digital skills education	Global
34	Identification of the determinants of rural digital divide, the attractiveness of rural areas, and opportunities for strengthening local governance	EU
35	Economic and social impact of digitalisation in rural areas	EU
*Inadequate device access and connectivity*		
36	Analysing the persistence of the digital divide in developed contexts	Netherlands
37	Exploring technology use and non-use in later life	Austria
38	Analysis of the gender gap in digital Skills	Greece
39	Assessing the digital divide between and within the 28 member-states of the European Union	EU
**Level 2: Digitalisation of ecosystems**		
*Financial exclusion*		
40	The position of women in agriculture at local level	Greece
41	Technical guidance note on rural women and financial inclusion	Global
*Market access constraints*		
7	Addressing gender barriers in agriculture innovation	Lithuania
42	The work of farmers in short food supply chains	Global
43	Assessing the gender dimensions of integration and economic and social upgrading in global value chains	Global
*Gender-blind design of technologies*		
2	Identification of patterns of inequality in digital agriculture	EU
44	Analysis of the gender-blind perspectives in the context of digitalisation and Work	Germany
45	Designing gender-inclusive digital solutions for agricultural development	Global
46	Building a bridge between debates on welfare and gender on one hand, and the social impact of algorithms on the other	Global
47	Exploring barriers, opportunities, and good practices for gender equality in entrepreneurship and innovation	EU
*Safety and trust issues*		
48	Analysis of European criminal law provisions for gender-based violence against women, including domestic and online violence	EU
49	Examining the shortcomings of the current approaches to law and policy, highlighting the failure to address gender inequality at the European supranational level	EU
50	Report on gender equality in the EU	EU
**Level 3: Digitalisation of the socio-ecological systems**		
*Data disempowerment*		
1	Assessing the EU agriculture policies in terms of integration of gender equality principles	EU
4	Understanding EU policies on agriculture and gender equality	EU
13	A global overview of gender inclusive tech	Global
51	Identification of actors who drive digital revolution in agriculture	Global
52	Analysis of the politics of digital agricultural technologies	Global
53	Analysis of the legal, technical, infrastructural, educational, financial and market challenges that can hinder or impose barriers to the digitalisation of agriculture	Spain
54	Exploring the perceived risks and benefits arising from the development of smart farming	Ireland
55	Analysing the effects on gender equality when the male-dominated bioeconomy sector merges with the male-dominated tech sector	Nordic countries
56	Digital government in the decade of action for sustainable development	Global
57	Analysis of how rural policy constructs gender inequality problems and gendered subjects	Sweden
58	Identification of how data-driven agricultural business models lock farm data into machines and devices, reducing downstream agricultural services market competitiveness	EU/USA
59	Analysing the socio-technical barriers hindering the widespread adoption and full potential of digital agriculture	Global
60	Reflections on the EU code of conduct for agricultural data sharing	EU

### Barriers at the level of digitisation of business processes

These barriers affect farm-level operations.

***Socio-cultural norms.*** Despite decades of gradual change, deep-rooted perceptions continue to cast farming as an inherently male domain, shaping the everyday experiences, opportunities, and limitations of women across rural Europe ^20^. This is particularly evident in the persistent stereotype that a ‘real farmer’ is a man, a cultural notion that exerts influence from language and public imagery, right through to the rules and practices governing land inheritance, policy formulation, and the distribution of both material and symbolic resources within rural communities. A compelling example of this phenomenon is evident in the Irish context, where only 13% of farm owners are female, despite women’s substantial contribution to daily farm management ^20^. The root causes are not simply legislative but are instead embedded in broader patriarchal traditions that position land ownership as a male prerogative and integrate land with masculine identity, authority, and continuity. This demonstrates how, even in the face of contemporary equality initiatives, cultural expectations still push families to prioritise sons over daughters as heirs, perpetuating the gender asset gap and reinforcing the symbolic association of ‘the farmer’ with maleness. These practices have critical ramifications, as ownership is often a prerequisite for access to credit, agricultural innovation programs, and participation in decision-making bodies. The everyday impacts of these norms extend far beyond issues of ownership. Women in agriculture often bear the double burden of labour, balancing unpaid, frequently unrecognised farm work with household and caregiving responsibilities ^6, 21^. Empirical research from Greece demonstrates that, despite an increase in women's formal participation, the hierarchical structure of rural life and agricultural labour remains largely resistant to significant change. Life stories of women farmers indicate that while perceptions of self-identity are evolving and some women are engaging in farming through new, non-traditional avenues, they continue to grapple with gendered expectations regarding appropriate tasks, as well as limitations on when and where they can work, and what qualifies as legitimate authority or expertise ^21^.

These constraints are further intensified by institutional barriers such as extension services and capacity-building workshops that are often scheduled at inconvenient times or presented in formats that are inaccessible to women managing family responsibilities. The scarcity of female extension agents can discourage women from seeking technical support, thereby intensifying their marginalisation ^22^. Notably, these socio-cultural patterns are reinforced through educational systems and socialisation processes from an early age. Gender-biased pedagogies and a lack of visible female role models in agricultural and STEM fields undermine young women’s aspirations and confidence. For instance, a study conducted in Moldova revealed that over 30% of girls who initially showed interest in information and communication technologies (ICT) ultimately abandoned their ambitions, citing the widespread belief that such careers were unsuitable for girls ^23^. This observation resonates across Europe, where, despite policy commitments to gender equality, cultural narratives regarding ‘appropriate’ work for women continue to influence educational and professional choices, particularly in rural areas ^24^

While evidence suggests that younger generations of both men and women exhibit greater intolerance for open discrimination ^22^, cultural norms surrounding farming and gender remain notably adaptable. These norms can integrate increased female labour without diminishing male dominance over ownership, recognition, or authority, a form of ‘passive feminisation’ that permits participation without meaningful power-sharing ^21^. Consequently, superficial changes in participation may mask the persistence of deeper inequalities. To achieve true gender equity in European agriculture, it is essential to address not only formal policies and practices but also the more subtle cultural logics that underpin them.

***Time and labour burden.*** Evidence from recent research and policy reviews demonstrates that rural women bear a disproportionate share of the total work burden ^1, 4^. They are not only critical contributors to agricultural production, constituting approximately 35% of the agricultural workforce, but are also predominantly engaged in unpaid or part-time roles that remain largely unrecognised and uncompensated in official statistics ^6^. Central to this discussion is the concept of ‘time poverty,’ as defined by the European Institute for Gender Equality (EIGE). Women in rural areas consistently perform more unpaid work encompassing childcare, elder care, cooking, and other domestic duties ^25^. This imbalance has far-reaching consequences; it constrains women’s ability to engage in paid employment, pursue training, or participate in public life. Consequently, women’s opportunities for professional advancement, entrepreneurship, and leadership in rural communities are severely limited, reinforcing the structural marginalisation of women in these settings.

For instance, the European Parliament ^26^ acknowledged that only 30% of women are categorised as farm holders, despite their active participation in farm management and decision-making. Most are instead classified as family workers, a designation that restricts their access to independent income, social protection, and pension rights, thereby perpetuating cycles of economic insecurity and dependence. As Shortall and Marangudakis ^27^ argue, a key reason for the lack of progress lies in how agriculture is conceptualised at the European level, primarily as a sector concerned with land, production, and subsidy, rather than as an occupation involving a diverse, gendered workforce. This sectoral framing means that Common Agricultural Policy (CAP) payments and support are distributed on the basis of land ownership and farm size, criteria that systematically favour men. The majority of large, profitable holdings are male-owned, while women are more likely to own or manage smaller, often subsistence-level farms, many of which are excluded from meaningful support.

The invisibility of women’s labour is further compounded by traditional gender norms and institutional inertia. Societal expectations around gendered divisions of labour not only influence the types of tasks women and men perform, assigning women to more manual, repetitive, and lower-status work, but also shape the allocation of time and resources within households. Comparative studies of time use in Europe reveal that countries with more traditional norms exhibit greater gender disparities in both paid and unpaid work. Where progressive family-friendly policies are implemented and where women’s participation in political and economic decision-making is higher, these gaps tend to be narrower ^28^. However, such positive examples remain the exception rather than the rule, with significant cross-country heterogeneity persisting across the EU.

Labour market segmentation worsens this gender gap. While increasing female labour force participation has been a central policy objective, this alone has not translated into economic empowerment. Instead, the expansion of part-time and precarious employment for women has often led to increased rates of in-work poverty, as detailed by Filandri and Struffolino ^29^. Their analysis shows that high female employment rates, in the absence of job quality improvements and social protection, do not guarantee a reduction in poverty or greater financial security for women. Rather, involuntary part-time work and low-wage employment are closely linked to household-level poverty, particularly where welfare states have retreated and labour markets have become more deregulated.

***Limited access to information.*** Despite the increasing involvement of women in agriculture and their clear need for technical and business knowledge, they continue to experience systemic exclusion from essential extension services and advisory platforms. This exclusion is not an isolated issue; rather, it results from a complex interplay of structural, cultural, and institutional barriers that collectively hinder women's ability to fully engage in agricultural development. Quantitative evidence from Amato *et al*. ^30^ indicates that this disparity in access to resources such as information is a significant contributor to lower entrepreneurial income among women farm holders, particularly at higher income levels. Furthermore, this disparity is perpetuated by the enduring presence of traditional family farming structures in countries like Italy, which often confine women to less profitable, secondary roles. This marginalisation inherently limits their access to essential extension services and information, reinforcing the cycle of lower income.

***Low digital literacy and confidence.*** While younger generations in the EU exhibit a narrowing gender gap in basic digital skills, it's important to note that this progress is overshadowed by the multifaceted digital exclusion that significantly affects older rural women, particularly those over the age of 45 engaged in agriculture ^31^. This divergence underscores a significant paradox as targeted EU strategies, while yielding modest gains among youth, are failing to address the systemic nature of digital disparities experienced by older women in rural settings. The roots of this disparity lie in a confluence of structural barriers. Foremost is the lower level of formal education prevalent among older rural women, especially in Southern and Eastern Europe ^32^. Educational attainment is a strong predictor of digital proficiency. Women with only primary education are significantly less likely to use the internet or digital tools, while those with secondary education are six times more likely to engage digitally ^33^.

Rural infrastructural weaknesses, including limited access to advanced training institutions, outdated broadband connectivity, and the demographic challenges of aging populations and youth outmigration compound these educational deficits. This creates a vicious cycle where limited initial opportunities for skill acquisition perpetuate socio-economic disadvantage and digital exclusion across generations ^34^. Beyond education and infrastructure, deeply ingrained stereotypes position technology as a male domain, eroding women's confidence, particularly regarding complex digital tasks like online financial management or farm planning software. Research, including findings from the Horizon 2020 DESIRA project indicates that while rural women adeptly use digital tools tailored to small-scale farming needs, they face significant hesitation with more advanced applications ^35^. This lack of confidence is frequently exacerbated by digital interfaces laden with technical jargon or available only in dominant national languages, failing to accommodate diverse literacy levels or linguistic minorities. Consequently, women are 25% less likely than men to use digital technologies for fundamental tasks ^33^.

***Inadequate device access and connectivity.*** Despite widespread recognition of the digital divide, much of the policy and academic discourse still treats connectivity and device access in rural Europe as primarily a matter of technical infrastructure ^36^, thus neglecting the deeply embedded socio-economic and gendered dimensions of exclusion. A study by Gallistl *et al*. ^37^ conducted with older adults in Austria challenged this simplistic view by revealing that digital non-use is not merely the absence of technology, but rather a complex negotiated practice influenced by social, cultural, and generational expectations. This perspective highlights how older women actively engage with technology, often resisting or adapting it in ways that traditional surveys fail to capture. Yet, current digital policies typically emphasize connectivity metrics, neglecting the authentic experiences of exclusion that extend beyond simple coverage maps and broadband speeds. As a result, initiatives focused solely on enhancing rural broadband may inadvertently reinforce, rather than alleviate digital marginalisation.

In addition, Perifanou and Economides ^38^ highlight the long-standing power imbalances within rural households and communities, where device ownership and digital skills are viewed as scarce resources, typically allocated first to men or younger family members. The perception that rural women lack digital skills stems from these exclusionary structures, and addressing this issue requires more than simply training the women. It necessitates confronting deeply rooted patriarchal values that dictate who is allowed to access, own, or control a digital tool. Elena-Bucea *et al*. ^39^ further emphasise that the so-called affordability barrier is not only about the outright cost of devices or data plans, but about persistent income inequalities and precarious rural economies. Policy frameworks that focus on one-off device provision ignore the recurring expenses required to maintain connectivity, expenses that are particularly burdensome for single-income or female-headed rural households. The literature also fails to account for the social cost of exclusion. For instance, when women lack access, entire families and communities are deprived of their capacity to fully participate in agricultural innovation, e-government, and social life. This entrenches a cycle of poverty and marginalisation that is not addressed by market-driven approaches to digital inclusion.

Moreover, Van Deursen and Van Dijk ^36^ expose the limits of equating internet access with digital empowerment. As access becomes increasingly widespread, inequalities shift from basic connectivity to material divides, specifically the number, quality, and diversity of devices, and the ongoing costs of keeping them functional. This is particularly evident in rural Europe, where mobile-only households, often female-led, constitute a growing mobile underclass. Mobile devices, while valuable for basic communication, are structurally inadequate for the full spectrum of agricultural, administrative, and educational digital participation. They constrain users to passive or superficial online activities, while more advanced or productive uses (e.g., managing CAP applications, accessing detailed agricultural data, or participating in e-learning) remain out of reach. By failing to address this material disparity, digital policy risks creating a facade of inclusion that masks deeper, more insidious forms of exclusion.

### Barriers at the level of digitalisation of ecosystems

These systemic barriers stem from structural inequalities embedded in the digital ecosystem, determining women's ability to leverage digital technologies and tools for scaling production, accessing markets, or participating in agri-food value chains.

***Financial exclusion.*** A significant factor contributing to financial exclusion is the persistent disparity in asset ownership between men and women. Women typically own fewer productive assets, particularly land, which is often essential for accessing formal financial resources such as loans and insurance ^20^. Although there has been notable progress, including a slight increase in the number of female farm holders reflected in official statistics, these advancements are often superficial and fail to tackle the underlying mechanisms that perpetuate women’s marginalisation ^19^. Moreover, the reliance on quantitative indicators, such as the proportion of farms registered to women, can obscure the true nature of control and agency within these contexts.

As highlighted by Tsiaousi and Partalidou ^40^, many women listed as heads of farms are not the actual decision-makers but are instead placeholders to meet eligibility criteria for subsidies or to enhance household benefits. This phenomenon of statistical inclusion reinforces traditional power hierarchies rather than disrupt them, as real economic control and strategic farm management continue to reside primarily within male domains. A recent report on rural women and financial inclusion by Maftei ^41^ re-echoes this sentiment. Therefore, it is misleading to interpret the increasing numbers of female farm ownership as a sign of genuine empowerment, when in reality, women’s roles remain constrained by deeply entrenched socio-cultural norms. Moreover, these norms stand as a central obstacle that undermines even the most well-intentioned interventions.

Financial institutions compound these inequities by using risk assessment models that penalise women for circumstances largely beyond their control, such as lack of collateral or formal credit history. These models are not neutral but reflect and reinforce existing gender biases, systematically relegating female farmers to the periphery of financial services ^6^. Rather than challenging exclusionary norms, such practices render policy efforts ineffective, as the availability of credit on paper does not translate to meaningful access in practice ^1^.

***Market access constraints.*** Market constraints are driven by the disproportionate concentration of women in small-scale or subsistence farming ^21^. Data from Latvia and Lithuania, for instance, demonstrate that women are far more likely to manage smaller farms, which inherently limits their ability to generate marketable surpluses and thus to participate in more lucrative, high-value markets ^6^. This structural skew is a product of both tradition and policy, where patrilineal land inheritance and persistent gender norms restrict women’s access to land and resources, pushing them into the less profitable margins of the agricultural sector. This finding is not unique to the Baltic states, but echoes throughout the EU, where the prevalence of women-managed farms diminishes as farm scale and market orientation increase.

In addition, Women’s roles have historically been confined to local markets or household food production, with men dominating the more profitable spheres of external trade and large-scale market transactions ^61^. These gendered expectations are perpetuated by the unequal distribution of unpaid care work, as seen in Lithuania where women overwhelmingly shoulder the burden of childcare and domestic labour. This double burden limits their time, mobility, and capacity to build business networks or access distant markets, compounding the barriers to their market participation ^24^.

Economic barriers are closely intertwined with these social norms. The underrepresentation of women in producer organizations and cooperatives, which are vital for gaining market intelligence, accessing new technologies, and negotiating better prices further isolates women farmers from mainstream market channels ^19^. The lack of collective bargaining power and limited exposure to market information reinforce a cycle of low returns and weak negotiating positions. Moreover, gender-based prejudices among buyers and intermediaries often result in women’s products being undervalued, forcing them to accept lower farm-gate prices ^22^.

While short supply chains such as farmers’ markets and direct sales present some opportunity for women to circumvent traditional barriers and gain higher margins, these avenues come with their challenges. As documented in a recent systematic review by Dupé *et al*. ^42^, engagement in short food supply chains demands high levels of unpaid or under-remunerated labour and presents scalability issues, thus limiting the potential for substantial economic advancement. The labour-intensity and complex organisational requirements of these models may further discourage women who already face significant time constraints.

Importantly, these gendered market access constraints are not confined to the EU context but reflect broader global patterns. In global value chains, as shown by Bamber and Staritz ^43^, increased participation by women does not automatically translate into equality. Gendered segmentation persists, with women concentrated in lower value, lower paid roles, and facing ongoing challenges in accessing finance, information, and productive resources. Thus, economic upgrading in value chains does not necessarily lead to social upgrading or reduced gender gaps.

***Gender-blind design of technologies.*** Historically, the evolution of agricultural tools, digital platforms, and services has operated under the assumption of user neutrality, implicitly prioritising male experiences and bodies as the standard ^44^. This has led to the systemic exclusion and marginalisation of women at various levels of agricultural engagement. For example, ergonomic mismatches in farming equipment disproportionately impact women, rendering certain tasks more heavy or even hazardous for them, thus limiting their productivity and autonomy within agricultural systems ^45^.

The digitalisation of agriculture has introduced new complexities to this gender gap. Automated decision-making systems (ADMS) for credit scoring, resource allocation, and extension services have surged in recent years, promising both objectivity and efficiency ^13^. However, these systems frequently depend on datasets and operational frameworks that reflect and reinforce existing gender biases. For instance, credit algorithms trained primarily on data from male farmers may inadvertently disadvantage female applicants, perpetuating patterns of exclusion from financial resources. The lack of transparency in algorithmic decision-making exacerbates the issue, leaving women adversely affected by these decisions with limited recourse to challenge unfair or biased outcomes ^46^.

Underlying the technological challenges are broader structural and cultural factors that influence who participates in, and benefits from rural innovation ^45^. The underrepresentation of women in technology design, leadership, and decision-making roles leads to a persistent absence of gender-sensitive perspectives in product development. Without women’s input, technologies continue to marginalise female users, limiting their participation and leadership in future innovation cycles ^52^. Adding on to these design deficiencies is the wide spread lack of gender-disaggregated data ^2^. Without systematic investment in research that differentiates between the experiences, needs, and constraints of male and female users, it becomes quite challenging to create agricultural and digital tools that are truly inclusive. Moreover, smaller agritech enterprises in particular may lack the resources or incentives to gather and analyse such data, resulting in an increase of neutral solutions that fail to meet the diverse needs of rural populations ^13^. Even when there is an awareness of gender issues, the absence of robust data impedes efforts to develop tailored interventions, training materials, or support services that effectively address the specific needs and contexts of women ^47, 53^.

***Safety and trust issues.*** The benefits of digitalisation are accompanied by significant safety and trust issues, particularly affecting women and girls. They are often vulnerable to online violence, harassment, and cyberstalking, which create substantial barriers to their meaningful engagement in digital agriculture and rural innovation ^54^. This issue is underscored by recent EU data revealing that one in ten women in the EU has experienced some form of cyber violence since the age of 15 ^48^. Moreover, this statistic may not fully capture the true extent of the problem due to the stigma surrounding these experiences and the tendency for underreporting.

While digital engagement in rural communities, such as participation in agricultural forums and professional social networks has become increasingly essential for success, many women find these spaces to be hostile and unsafe. As Barker and Jurasz ^49^ point out, digital platforms that are meant to empower can instead foster environments of intimidation, abuse, and gendered silencing. These factors significantly undermine women's confidence in utilising digital tools for business, education, or leadership within their communities. Consequently, such dynamics risk reinforcing offline inequalities and perpetuating traditional gender hierarchies, even as rural societies experience modernisation.

The persistence and evolution of these risks are significantly influenced by both technological advancements and enduring social structures. While the European Union has made notable strides such as the 2024 Directive aimed at combating violence against women, which criminalises various forms of cyber violence, considerable gaps still exist in the recognition and enforcement of these standards at the national level ^50^. Many Member States lack comprehensive definitions of gender-based digital violence, often resorting to gender-neutral language in legislation that fails to acknowledge the disproportionate targeting of women and girls. This results in inconsistent protections, weak enforcement, and a lack of intersectional awareness, particularly disadvantaging rural women who may already face marginalisation due to other aspects of their identity, such as age, ethnicity, or immigrant status.

### Barriers at the level of digital transformation of socio-ecological systems

These barriers reflect structural and systemic inequalities tied to governance, data, and sustainability.

***Data disempowerment.*** The ongoing digital transformation of European agriculture, characterised by the integration of big data analytics, sensors, and AI-driven tools, has introduced new dynamics into an already complex gender landscape ^51, 53, 54^. While the potential of digital agriculture is often presented in terms of increased productivity, sustainability, and improved decision-making at the farm level, an expanding body of research highlights a persistent and deepening gender gap, particularly regarding data accessibility and digital empowerment ^4, 55, 56^. Data disempowerment involves not only women's inadequate access to digital tools but also their exclusion from agricultural data systems, statistics, and digital service provision ^13, 55^.

The underrepresentation of women in agricultural datasets stems from reporting practices and institutional limitations. Many women may identify themselves as helpers or classify their roles as not employed, while national statistical systems often fail to capture women’s part-time, seasonal, and unpaid agricultural contributions ^53^. This lack of accurate, sex-disaggregated data cascades through various layers of policy and innovation. Official statistics consistently underestimate women’s contributions, distorting their visibility and obscuring the realities of their roles in EU agri-food systems ^1^. Policymakers and planners, relying on gender-blind datasets, may neglect or inadequately address the specific needs of women. Consequently, agricultural support, subsidies, and research initiatives often fail to adequately serve them ^4^. Evidence from policy analysis indicates that gender mainstreaming within the EU’s CAP and Rural Development Policy frequently amounts to little more than ‘lip service.’ Initiatives such as training, gender budgeting, and impact assessments tend to be superficial, with the prevailing policy narrative continuing to position the male farmer as the norm ^57^. Additionally, the absence of robust monitoring and accountability frameworks further entrenches these structural barriers.

Similar patterns can be observed in the private sector, where agritech companies and digital solution providers create products and platforms ranging from farm management applications to credit scoring and insurance tools that predominantly reflect male farming practices ^13, 58^. This design bias is further compounded by the lack of detailed, gender-specific data on women's agricultural work, resulting in digital solutions that are often less relevant, accessible, or user-friendly for women farmers. Consequently, the adoption of digital tools among women remains low, creating a feedback loop where limited data generation from women's operations restricts their access to precision advice, tailored financial products, and digital innovations ^55^.

Beyond issues of access and representation, the disempowerment of data in EU agriculture also raises concerns about agency and rights. As digitalisation progresses, the value of farm-level data increases. However, frameworks governing data rights, privacy, and consent frequently overlook the specific vulnerabilities faced by women, especially those with lower digital literacy or limited decision-making power ^52, 59^. Women may inadvertently surrender control over their farm data as a condition for accessing services, often without fully understanding the implications or receiving equitable benefits ^60^. This situation exacerbates their exclusion, reinforcing both underrepresentation and a lack of agency within the rapidly evolving digital agricultural economy.

## Harnessing the potential of digital technologies in levelling the gender gaps

Harnessing digitalisation to tackle deep-rooted gender barriers in EU agriculture offers exciting possibilities but also brings significant challenges. While digital transformation holds the potential to disrupt traditional power structures, its effectiveness depends on how interventions are designed, implemented, and situated within broader socio-cultural and institutional contexts. If digital interventions are not carefully planned with an awareness of gender issues and if they lack strong policy support, there is a real risk they could actually make inequalities worse, even while appearing to make progress ^1, 6^.

One step toward advancing gender equality lies in increasing women’s visibility and agency through digital platforms. Online forums, social media campaigns, and e-learning initiatives not only facilitate access to information and resources but also serve as powerful platforms for challenging the idea that farmers are typically male ^20, 24^. However, these digital platforms can only truly make a difference if they reach women who are often left behind, especially those who are not confident with technology or who are busy with household responsibilities. Flexible, women-focused online services, and peer networks can help include more women ^22^, but their impact is still limited by ongoing digital gaps. This is especially true for older women in rural areas ^37^. Moreover, the tendency of digital campaigns to attract primarily those already inclined towards innovation risks deepening, rather than bridging, gender gaps if complementary offline strategies are neglected.

The implementation of labour-saving digital tools and remote advisory services represents a promising approach for alleviating the time poverty that characterises women’s agricultural and domestic workloads ^25, 28^. By making tasks more efficient and allowing women to connect with markets from a distance, these technologies have the potential to boost women’s independence and earning power. However, such benefits are dependent on the quality of the technology, the relevance of content, and the ease of device access and connectivity. For instance, one-off device provision schemes are unlikely to succeed unless they are accompanied by ongoing support, affordable data plans, and a focus on the family dynamics that influence who gets to use technology and how ^37, 39^.

Addressing systemic barriers at the digital ecosystem level holds significant potential to advance women's financial inclusion and market participation by improving their access to credit, insurance, and e-commerce ^6^. However, persistent challenges such as collateral requirements, land ownership constraints, and hidden algorithmic biases can still pose significant obstacles. Therefore, achieving genuine inclusion requires combining digital finance solutions with broader structural reforms. These reforms should include transparent and participatory algorithm design and the systematic integration of gender-disaggregated data to prevent digital tools from replicating the very biases they aim to overcome ^47^. Similarly, while e-commerce platforms and digital cooperatives can enhance women's market access and collective bargaining power, they too are not complete solutions on their own.

Additionally, building safe and trusted digital ecosystems is paramount to empowering rural women, enabling their full participation in agricultural innovation and rural leadership ^48, 54^. This requires a dual approach: first, the consistent and robust enforcement of EU directives that criminalise gender-based cyber violence across all Member States ^50^. Second, it necessitates the proactive design of inclusive online platforms featuring strong moderation, clear safety protocols, and community guidelines developed with an intersectional lens ^49^. By combining rigorous legal protection with intentionally safe digital spaces, online harassment can be effectively mitigated to ensure that digital platforms become engines of opportunity for rural women.

Furthermore, in order to achieve change at the level of socio-ecological system, data governance frameworks should be redesigned to guarantee that women's contributions are recognised, their data rights are upheld, and their agency is honoured. Participatory research and inclusive policymaking are essential for the co-creation of digital agricultural solutions that reflect and respond to the real needs of diverse women ^53^.

### Practical cases from CODECS living labs across Europe

***Digitisation of business processes level.*** The implementation of automation and precision agriculture technologies within European Living Labs is significantly altering the temporal and structural dynamics of agricultural work. Technologies such as the Farm Management Information System (FMIS) in Italy, orchard management systems in the Czech Republic, and smart irrigation platforms in Slovakia alleviate time and labour burdens by reducing reliance on manual labour. This enhancement in process efficiency and resource optimisation directly decreases the physical effort and time expenditure required of workers. Consequently, women are afforded the opportunity to redirect their time toward professional development, off-farm income-generating activities, or enhanced decision-making roles. Thus, automation functions not only as a driver of productivity but also as a catalyst for redefining time allocation in ways that empower women's autonomy and agency within the sector ^62^.

Similarly, digital tools are addressing the structural invisibility of women's contributions. As demonstrated by the RAMAS living lab in Macedonia, digital records and farm management platforms enable women to formally document their agricultural activities. This generates verifiable data trails that are critical for facilitating access to credit, social security benefits, and targeted subsidies. When equipped with accessible interfaces and mobile applications, these platforms allow women to transition from being overlooked contributors to recognised economic agents, further solidifying their empowered status ^20^.

***Digitalisation at the digital ecosystem level.*** CODECS Living Labs are demonstrating that digitally enabled agricultural tools, when designed with intentionality, can significantly expand women's access to vital agronomic and weather information. Initiatives in Serbia and Spain, for instance, utilise IoT-enabled platforms to generate real-time data on microclimates, soil conditions, and disease risks. The critical innovation lies in connecting these data streams with accessible delivery mechanisms. By integrating voice-based advisories, visual dashboards, and women-only digital spaces, drawing from models like Slovenia’s LoRaWAN network and Poland’s APPETIT platform, these tools successfully bypass prevalent literacy barriers and social restrictions. Such inclusive design principles directly counter low digital confidence, allowing women to build technological fluency within supportive and familiar environments ^63^.

Furthermore, to address systemic financial exclusion and barriers to market access, some living labs are pioneering digital marketplaces and traceability solutions. Blockchain-based tools and open-source platforms exemplified by Cloughjordan’s food hub in Ireland enhance supply chain transparency and promote a more equitable distribution of value. The efficacy of these platforms is markedly increased when they are coupled with low-bandwidth applications and multilingual interfaces. This combination significantly improves women's access to formal markets, enabling direct-to-consumer sales or participation in certified supply chains. Consequently, these digital pathways strengthen the bargaining power and income of smallholders, particularly women who are frequently marginalised by traditional market structures ^64^.

Beyond transactional and informational tools, digital platforms are also fostering social capital through peer-to-peer learning and collaborative marketing. Living Labs, such as the Scottish Small Farms and Digital Platforms initiative, leverage social media and collaborative platforms to create virtual communities. These spaces, designed with embedded safeguards such as encrypted communications, the inclusion of female mentors, and actively moderated communities, establish trusted digital environments that mitigate safety concerns. For women historically excluded from male-dominated agricultural forums and extension services, these platforms provide an empowering alternative. They serve as vital spaces to access support, share knowledge, and engage in collective advocacy, thereby challenging existing patterns of exclusion and building robust networks of solidarity ^65^.

***Digitalisation at the socio-ecological system.*** This highest level of digitalisation is characterised by interventions that fundamentally reshape institutional norms, governance, and design processes. The participatory model of the Living Labs, where women engage as both testers and co-designers, presents a structural shift from gender-blind innovation to inclusive design. Integrating feedback from women farmers during the development phase ensures that digital solutions are not just available but genuinely usable and beneficial. This co-creation process embeds gender sensitivity into the very architecture of innovation, from interface design to data input mechanisms, setting a standard for equitable technology development across the EU ^66^.

## Policy recommendations

Building upon the multi-level CODECS framework and the evidence presented, this section proposes policy recommendations to advance gender equality in EU agriculture and rural areas through equitable digitalisation.

### Enhancing gender equality at the business process level

***Integrate gender sensitivity into digital literacy and capacity building.*** Policymakers at the national and EU levels should develop and include gender-specific digital skilling programmes, tailored to the needs of rural women across age groups and educational levels. The initiatives should prioritise older women and those with little formal education, using context-appropriate training formats, local language materials, and flexible delivery methods ^67^. Collaboration with local women’s organisations, agricultural extension services, and educational institutions is essential for effective outreach. In addition, the involvement of mentors and female role models can promote trust and acceptance in rural communities.

***Facilitate equitable access to digital devices and connectivity.*** Targeted subsidies and financial support for digital device acquisition and broadband access should be made available to women in rural and agricultural settings, especially female-headed households. Public-private partnerships should be incentivised to develop and scale mobile-first solutions (e.g., SMS, voice-based services) that are suitable for basic-level devices and varying literacy levels. Policy makers should ensure that digital inclusion efforts go beyond basic connectivity to consider sustainable affordability and usability ^53^.

***Formal recognition of women’s labour.*** Statistical and administrative protocols should be reformed to recognise both paid and unpaid labour contributions by women in agricultural systems. Mandating the use of digital farm management platforms that record women’s roles and outputs can facilitate access to credit, social protection, and agricultural subsidies. It is also essential that all agricultural data collections produce and report sex-disaggregated statistics to enable evidence-based policy ^1^.

### Fostering gender equality within the digital ecosystems

***Promote gender-responsive financial innovation.*** Financial inclusion should be improved by redesigning digital credit, insurance, and payment platforms that take into account the specific constraints faced by rural women, such as limited collateral and informal credit histories. Regulators should require a gender review of algorithms used in digital finance, to ensure that automated systems do not reproduce biases. Collaboration with women’s cooperatives and rural financial institutions should be prioritised in the co-development of tailored digital financial products ^41^.

***Strengthen market access and entrepreneurship.*** Support for women’s participation in digital value chains needs to be scaled up by providing funding, technical assistance, and capacity-building for women-led cooperatives, e-commerce initiatives, and digital marketplaces. Technology developers and agribusiness platforms should be required to consult and involve women users throughout product development and piloting to ensure usability and inclusivity ^64^. Targeted support should be provided for digital competences, marketing skills, and collective bargaining to enable women to navigate and succeed in digital markets.

***Ensure safe, inclusive, and empowering digital spaces.*** Digital agricultural platforms need to enforce robust online safety standards, including proactive moderation, transparent reporting mechanisms and explicit anti-harassment policies. It is recommended to invest in the creation and support of women-only or women-led digital communities, including forums and mentorship networks, to create safe spaces for knowledge sharing, leadership and advocacy ^68^.

### Advancing structural transformation at the socio-ecological system level

***Institutionalise gender-responsive co-design and innovation.*** Participatory co-design approaches, such as those demonstrated in CODECS Living Labs, should be institutionalised as a standard practice in all publicly funded digital agricultural innovation projects. The active participation of women as co-creators and end-users is crucial for the relevance, acceptance, and impact of new technologies ^69^. Concurrently, guidelines for gender-responsive digital innovation should be developed at both the national and EU-level, and compliance with these guidelines made a prerequisite for funding.

***Reform data governance for equity, agency, and accountability.*** Policymakers should mandate the systematic collection and publication of sex-disaggregated data and require regular gender impact assessments for all digital agricultural interventions. Additionally, data rights frameworks should empower women to control and benefit from agricultural data generated by and about them, with explicit consent protocols and transparency. Further, the establishment of independent monitoring bodies to oversee adherence to gender equity standards in digital agriculture is strongly recommended ^1^.

***Ensure policy coherence and long-term sustainability.*** Digital agriculture strategies should be aligned with existing frameworks for gender equality, rural development and the Sustainable Development Goals (SDGs). Moreso, cross-sectoral working groups, integrated funding streams and harmonised monitoring and evaluation mechanisms will be essential for effective and sustainable impact ^13^. We further recommend that policies are regularly reviewed and updated based on empirical evidence and stakeholder feedback.

## Conclusion

Digitalisation presents a powerful yet double-edged opportunity for addressing entrenched gender inequalities in EU agriculture. While emerging technologies can disrupt existing hierarchies and enable more inclusive participation, they may also reinforce exclusion if developed without attention to the socio-cultural and institutional barriers women face. The CODECS framework has shown that structural gender disparities manifest at all levels of digital transformation, from individual farms to the broader socio-ecological systems shaping rural life. Overcoming these challenges requires more than technical solutions, it demands integrated strategies that centre women as agents of change, not passive recipients of innovation. Through participatory design, gender-disaggregated data collection, inclusive governance, and context-sensitive digital technologies and tools, it is possible to build digital ecosystems that not only reduce gender gaps but genuinely promote equity. The CODECS Living Labs across Europe offer promising examples of how digital technologies can be harnessed to empower rural women, whether through automation that reduces labour burdens, platforms that enhance market access, or safe digital spaces that foster learning and solidarity.

Ultimately, to realise the transformative potential of digitalisation, EU policymakers and stakeholders should include gender equality into every layer of the digital agricultural agenda. This includes reforming data governance frameworks, ensuring accountability in digital policy, and supporting co-creation processes that elevate women’s voices and leadership. Only through such comprehensive and intersectional approaches can digitalisation become a genuine driver of inclusive and sustainable rural development.

## Ethics and consent statement

Ethical approval and consent were not required.

## Data Availability

No raw data associated with this article. All the materials used are either published peer reviewed articles, policy documents, technical reports or even Living Labs reports (these are published on the CODECS website). All of which have been cited.
