# Leveraging digital solutions to address gender inequalities in EU agriculture: a CODECS framework application

**DOI:** 10.12688/openreseurope.21445.2

**Published:** 2026-04-11

**Authors:** Barbra Comfort Akello, Gianluca Brunori

**Affiliations:** 1 Department of Agricultural, Food and Agro-Environmental Sciences, University of Pisa, Pisa, 56124, Italy

**Keywords:** Gender inequality, Agriculture, Digitalisation, CODECS, EU policies

## Abstract

**Background:**

Despite longstanding EU commitments to gender equality, structural disparities continue to restrict women’s participation and leadership in agriculture and rural development. Digitalisation is increasingly promoted as a catalyst for addressing these inequalities. However, there is a limited holistic understanding of the conditions under which digital innovations can foster inclusive and sustainable outcomes. A key barrier is the absence of integrative frameworks that can systematically categorise the multifaceted evidence emerging from digital transitions. This limitation hampers both the comparability of findings and their practical relevance for policymaking. To address this, the study employs the CODECS conceptual framework to evaluate how digitalisation intersects with gender inequality in EU agriculture.

**Methods:**

Drawing on peer-reviewed literature, policy analyses, technical reports and insights from CODECS Living Labs, the study explores dynamics across the three levels of business process digitisation, ecosystem-wide digitalisation, and broader socio-ecological systems transformations. Results The analysis shows key persistent gaps, including gender-biased technology design, socio-cultural norms, low digital confidence, financial exclusion, and data disempowerment. However, it also identifies opportunities, particularly when digital tools are designed with context-awareness and co-created through participatory approaches. Living Labs demonstrate how innovations such as accessible advisory services, digital marketplaces, and moderated learning platforms can empower rural women by increasing their visibility, agency, and access to resources.

**Conclusions:**

Digital technologies alone cannot overcome entrenched inequalities. Promoting equity requires complementary reforms in governance, institutional structures, and data accountability. The CODECS framework provides a structured lens for understanding and addressing gendered dynamics in agricultural digitalisation, offering practical insights for inclusive innovation and evidence-based policymaking in Europe.

## Plain Language Summary

In rural Europe, women in farming face significant challenges. Despite carrying out a large portion of the agricultural work, they often have less access to land, income, education, and decision-making power than men. This inequality makes it more difficult for women to operate successful farms, earn a fair living, or have a strong voice in their communities. This study looks at whether digital technologies such as soil sensors, crop-monitoring drones, and digital marketplaces could help reduce these gender gaps.

To investigate this, the authors use the multi-level CODECS framework that looks at the interactions between day-to-day farm work, the surrounding digital services and markets, and broader national policies and social norms. This approach helps reveal how gender inequalities appear at each of these levels and helps identify ways to address them. The research highlights real-world examples from across Europe where digital platforms have successfully helped women farmers by increasing their visibility, providing access to new markets, saving them time, and building supportive networks. However, it also cautions that if digital tools are not designed with women’s needs in mind, they could unintentionally make existing inequalities worse. Finally, the study offers practical policy suggestions, such as using co-design strategies that include women, improving data collection on women in agriculture, and ensuring that online spaces are both safe and accessible.

## Introduction

Agriculture in the European Union (EU) remains characterised by deep-rooted gender inequalities, despite decades of political efforts to achieve equality. ^1^ Women continue to face significant barriers in accessing land, finance, education, technology, as well as meaningful decision-making power, leaving them at a disadvantage compared to men in almost all areas of agriculture and rural life. ^2^ Although gender equality is frequently cited in rural development objectives, many initiatives fail to address the underlying socio-cultural structures that reinforce this marginalisation. ^3^ As Shortall and Marangudakis ^4^ highlight, a fundamental structural imbalance of influence underpins the ineffectiveness of these measures. Dominant agricultural lobbying groups wield substantial influence over EU policy priorities, while gender-focused advocacy remains comparatively under-resourced and lacks robust legal and institutional backing. As a result, gender concerns are systematically sidelined in EU’s agricultural policy framework.

The outcome of this neglect is adverse for women in rural areas, contributing to lower productivity, fewer entrepreneurial opportunities, limited economic independence, and diminished quality of life. ^1^ ^,^ ^4^ This marginalisation is further evidenced by the reality that although women make up 35% of the agricultural labour force in the EU and contribute 31% of total working hours to the economy, their work continues to be undervalued, and their specific needs often remain unaddressed in policy and practice. ^5^ The persistence of these gaps contrasts with the EU’s long-standing commitment to gender equality, enshrined in its treaties and reinforced by milestones such as the 1957 Treaty of Rome, the 1996 communication on gender mainstreaming, and the Gender Equality Strategy 2020–2025. ^6^

From a gender and development perspective, this problem is best understood through the lens of empowerment, rather than representation or resource allocation alone. ^7^ Empowerment is conceptualised here as a process through which individuals gain the ability to make strategic life choices in contexts where such ability has been systematically constrained. ^8^ This process unfolds across interconnected dimensions, including access to resources, the exercise of agency, and the institutional conditions that enable or restrict the translation of resources and agency into sustained outcomes. Persistent gender inequalities in agriculture therefore reflect not only disparities in assets or opportunities, but enduring limitations on women’s capacity to influence decisions affecting their labour, livelihoods, and futures. ^9^

Digitalisation in agriculture is widely promoted in policy and industry circles as a strategic opportunity to redress the structural imbalances. ^10^ Narratives associated with this so-called fourth agricultural revolution portray digital technologies such as artificial intelligence (AI), blockchain, and the Internet of Things (IoT) as neutral tools that can reduce labour burdens, improve access to information and markets, and enhance productivity. ^11^ ^–^ ^13^ However, such techno-optimist accounts risk obscuring the social and institutional conditions under which digitalisation unfolds. Digital technologies do not operate in a vacuum as they are embedded in existing socio-technical systems and shaped by prevailing norms, power relations, and governance arrangements. ^11^ Consequently, while digitalisation may expand women’s resources and agency, it may also reproduce or intensify gender inequalities by reinforcing existing patterns of exclusion in technologically mediated forms.

Moreover, contemporary digital transformation differs fundamentally from earlier waves of technological change in agriculture, with distinct implications for gender relations. Historical mechanical innovations were largely characterised by a substitution effect, whereby machines replaced manual labour and directly reshaped rural labour markets. The post-war introduction of milking machines in Norway illustrates this dynamic clearly. Between 1930 and 1970, young rural women displaced from dairy farming were more likely to transition into better-paid, skilled employment, particularly in the public sector and to invest more in higher education. ^14^ Although men were also affected by mechanisation, their displacement was less pronounced, and they did not experience comparable occupational upgrading. This pattern reflects a gender-biased technological change that contributed to the masculinisation of Norwegian agriculture, while, paradoxically narrowing long-term gender gaps in employment and education. ^14^

The digital technologies shaping present day European agriculture, such as AI, data platforms, and IoT, function less as direct substitutes for labour and more as cognitive and institutional layers that mediate information flows, decision-making processes, and governance arrangements. ^12^ Rather than simply displacing work, digital systems influence who has access to data, whose knowledge is recognised, and who exercises authority within increasingly data-driven agricultural systems. As a result, digitalisation can generate divergent gendered outcomes. On the one hand, it may empower women by lowering physical barriers, expanding access to markets and knowledge, and enabling new forms of agency, including opportunities for innovation and change agency. ^6^ On the other hand, it may reinforce exclusion through biased data infrastructures, unequal digital skills, and male-dominated governance structures, thereby intensifying a triple divide based on gender, digital access, and rural location. ^11^ Recognising this shift from labour substitution to cognitive mediation is essential for understanding why digital transformation in agriculture does not produce uniform gender effects.

Current discussions on digital agriculture in the EU reflect this ambivalence. ^10^ ^,^ ^15^ ^,^ ^16^ While a growing body of literature documents the economic and environmental benefits of digital technologies, far fewer studies systematically evaluate their implications for gender equality. ^2^ Where gender is addressed, analyses often remain fragmented, focusing on isolated dimensions such as access to technology, labour participation, or entrepreneurship. ^2^ ^,^ ^4^ This has resulted in a lack of integrated explanations capable of clarifying how and under what conditions digitalisation of agriculture contributes to women’s empowerment, rather than merely describing its effects. ^11^ The absence of a coherent framework capable of linking digital transformation to power, agency, and institutional change limits the analytical and policy relevance of the existing evidence.

This study addresses this gap by employing an empowerment lens, operationalised through the framework developed in the Horizon project *Maximising the CO-benefits of agricultural Digitalisation through conducive digital ECoSystems* (CODECS). Instead of treating digitalisation as an isolated technological intervention, the analysis examines how empowerment processes develop within agricultural systems experiencing digital transformation. The CODECS framework conceptualises these systems across three interconnected levels: farm business processes, digital ecosystems, and broader socio-ecological systems. ^17^ This multi-level perspective enables the identification of how opportunities for empowerment are facilitated or restricted at various scales, and how digital transformation interacts with existing governance structures, norms, and power relations in European agriculture.

Guided by this integrated approach, we address two research questions:

1) What systemic gender-related barriers constrain women’s empowerment in EU agriculture?
2) Under what conditions can digital technologies and digital ecosystems contribute to gender-transformative change?

By conceptualising digitalisation as a contested process of empowerment rather than a neutral technological shift, the study advances debates in gender and development, digital inclusion, and sustainable agricultural transitions. It contributes a theoretically grounded synthesis that highlights why digitalisation matters not only for productivity and innovation, but for the redistribution of power within European agriculture.

## Theoretical framework

This study adopts an empowerment-based perspective to analyse how digitalisation reshapes gender relations in EU agriculture. It draws on the framework developed by Kabeer, ^8^ which conceptualises empowerment as a process through which individuals gain the capacity to make strategic life choices in contexts where this ability has been systematically constrained. This approach is particularly suited to the analysis of gender inequality in agriculture, as it moves beyond descriptive accounts of participation to examine the underlying power relations that structure access to resources, decision-making, and outcomes.

Kabeer’s framework distinguishes between three interrelated dimensions of resources, agency, and achievements. Resources encompass not only material assets such as land, capital, and technology, but also human and social resources, including skills, knowledge, and networks. Crucially, access to these resources is mediated by institutional rules and social norms, meaning that their distribution reflects existing power structures rather than neutral allocation mechanisms. Agency refers to the capacity to define goals and act upon them, extending beyond observable decision-making to include processes of negotiation, resistance, and the ability to envision alternative futures. Achievements represent the realised outcomes of these processes, indicating the extent to which individuals are able to translate resources and agency into meaningful improvements in their lives.

While widely applied in development studies, the framework requires adaptation to capture the specific dynamics of digital transformation in advanced agricultural systems. Kabeer’s model does not explicitly account for the ways in which digital technologies mediate access to resources, reshape forms of agency, and influence institutional structures. Digitalisation introduces new forms of power, including control over data, algorithmic decision-making, and platform-based coordination, which cannot be fully understood through traditional resource-based analyses alone. As such, this study extends the empowerment framework by explicitly incorporating digital infrastructures as sites where power is produced and exercised.

To operationalise this perspective, the study integrates Kabeer’s framework with the multi-level analytical structure developed in the CODECS project. ^17^ This integration addresses a key limitation of empowerment-based approaches, which often focus on individual or household-level dynamics while under-theorising the broader systems within which these processes unfold. The CODECS framework enables the analysis of digitalisation as a system-level transformation, linking micro-level experiences of empowerment to meso and macro-level institutional dynamics.

The analysis is structured across three interconnected levels.

Level 1: Digitisation of business processes

At this level, digitalisation affects the distribution of resources and the exercise of agency through access to technologies, digital skills, and decision-making authority. Gendered disparities at this level may manifest in unequal access to digital tools, limited participation in technology adoption decisions, and differential control over digitally mediated production processes. These dynamics directly influence empowerment outcomes, including productivity, income, and professional recognition.

Level 2: Digitalisation of ecosystems

At the ecosystem level, digitalisation shapes interactions among a broader set of actors, including advisory services, cooperatives, platforms, and markets. Here, power operates through network structures, institutional norms, and access to information flows. Participation in digital ecosystems can enable forms of collective agency and social capital accumulation but may also reinforce exclusion if governance structures remain male-dominated or if entry barriers such as digital literacy or platform access are unevenly distributed.

Level 3: Socio-ecological systems transformation

At this level, digitalisation is embedded within wider policy frameworks, regulatory regimes, and socio-economic institutions that shape the distribution of resources and opportunities. This includes control over data infrastructures, standard-setting processes, and the design of digital policies. These structures determine whether digital transformation reinforces existing gender inequalities or creates conditions for more transformative change by redistributing power and enabling new forms of participation.

By linking empowerment processes across these three levels, the framework enables a more comprehensive analysis of how digitalisation reshapes gendered power relations in agriculture.

## Methodological approach

This study employs a qualitative approach and draws on three complementary sources of qualitative evidence.

Peer-reviewed academic literature was used to establish the conceptual and empirical foundations of the analysis. Relevant studies were identified through targeted searches of major academic databases, such as Google Scholar, Web of Science, and Scopus, and through snowballing citation tracking. Source selection was based on conceptual relevance to gender, agriculture, rural development, and digitalisation, rather than exhaustive coverage. Publications from 2010 onwards were prioritised to reflect developments in digital agriculture, while earlier foundational works were included as needed for theoretical context. Only studies focusing on European countries or comparable developed-country contexts were used to ensure contextual relevance.

In addition, policy and institutional documents, including EU strategies, parliamentary reports, gender equality assessments, and publications from international organisations, were used. These materials were sourced from official EU repositories, institutional databases, and project websites. Documents were selected for their relevance to agricultural policy, rural development, digital transformation strategies, and gender equality initiatives, with particular attention to those highlighting governance structures, institutional priorities, and policy frameworks influencing digitalisation in agriculture.

Furthermore, insights were obtained from documented outputs of the CODECS Living Labs, including project reports, technical deliverables, and publicly available summaries. From the full set of 20 Living Labs, two cases were selected based on their explicit engagement with gender-related issues, to offer illustrative insights into how these dynamics may manifest in practice, rather than providing comprehensive case analysis.

All data were analysed using a deductive coding approach aligned with the three analytical levels of the CODECS framework. Identified barriers and enabling conditions were assigned to levels according to their primary locus of influence, including operational access and decision-making at the farm level (Level 1), participation in platforms, markets, advisory systems, and organisational networks (Level 2), and structural power relations embedded in governance arrangements, data infrastructures, and institutional leadership (Level 3). Cross-level interactions and feedback effects were documented when issues spanned multiple levels.

## Barriers to gender inclusivity in agriculture

Gender inequality in EU agriculture arises from interlocking mechanisms operating across multiple levels of the agricultural system, rather than from isolated constraints. These mechanisms influence women’s access to resources, their capacity to exercise agency, and their ability to achieve meaningful outcomes. Applying the CODECS framework (as summarised in Table 1), the analysis demonstrates how barriers are reproduced at the levels of farm practices, digital ecosystems, and broader governance structures. ^18^

**Table 1.** Key gender barriers in European agriculture mapped to the CODECS framework’s digitalisation levels.

Reference	Main area of contribution	Scope of the study
**Level 1: Digitisation of business processes**		
*Barriers related to socio-cultural norms*		
19	Female participation in farming and reasons for low rate of female farm ownership	Ireland
20	Depth of changes to contested gender identities in a (still) male dominated agricultural sector	Greece
21	Gender inequality in agriculture	Lithuania
22	Motivations and barriers for girls and women in STEM and ICT domains	Moldova
*Time and labour burden*		
5	Identification of differences between male and female operated farms	EU
23	Tracking progress against long-lasting challenges regarding gender equality	EU
24	Examining the mismatch between European legislation on employment and women’s employment position in agriculture	EU
25	Measuring gender gaps in both paid and unpaid work	EU
26	Gender differences in time use across age groups	Europe/America
27	Gender differences in time use over the life course	Europe
28	Understanding the role of labour market characteristics	EU
*Limited access to information*		
29	Analysis of the gender gap in entrepreneurial income within Italy’s farm sector	Italy
*Low digital literacy and confidence*		
31	Gender equality statistics in agricultural and digital sectors	EU
32	Analysing gaps in education levels between rural and urban areas	EU
33	Rationales and recommendations for gender equal digital skills education	Global
34	Identification of the determinants of rural digital divide, the attractiveness of rural areas, and opportunities for strengthening local governance	EU
35	Economic and social impact of digitalisation in rural areas	EU
*Inadequate device access and connectivity*		
37	Analysing the persistence of the digital divide in developed contexts	Netherlands
38	Exploring technology use and non-use in later life	Austria
39	Analysis of the gender gap in digital Skills	Greece
40	Assessing the digital divide between and within the 28 member-states of the European Union	EU
**Level 2: Digitalisation of ecosystems**		
*Financial exclusion*		
41	The position of women in agriculture at local level	Greece
43	Technical guidance note on rural women and financial inclusion	Global
*Market access constraints*		
6	Addressing gender barriers in agriculture innovation	Lithuania
46	The work of farmers in short food supply chains	Global
47	Assessing the gender dimensions of integration and economic and social upgrading in global value chains	Global
*Safety and trust issues*		
50	Analysis of European criminal law provisions for gender-based violence against women, including domestic and online violence	EU
49	Examining the shortcomings of the current approaches to law and policy, highlighting the failure to address gender inequality at the European supranational level	EU
51	Report on gender equality in the EU	EU
**Level 3: Digitalisation of the socio-ecological systems**		
*Data disempowerment*		
1	Assessing the EU agriculture policies in terms of integration of gender equality principles	EU
4	Understanding EU policies on agriculture and gender equality	EU
56	A global overview of gender inclusive tech	Global
55	Analysis of the politics of digital agricultural technologies	Global
57	Analysis of the legal, technical, infrastructural, educational, financial and market challenges that can hinder or impose barriers to the digitalisation of agriculture	Spain
48	Exploring the perceived risks and benefits arising from the development of smart farming	Ireland
54	Analysing the effects on gender equality when the male-dominated bioeconomy sector merges with the male-dominated tech sector	Nordic countries
61	Digital government in the decade of action for sustainable development	Global
63	Analysis of how rural policy constructs gender inequality problems and gendered subjects	Sweden
60	Analysing the socio-technical barriers hindering the widespread adoption and full potential of digital agriculture	Global
62	Reflections on the EU code of conduct for agricultural data sharing	EU
*Gender-blind design of technologies*		
2	Identification of patterns of inequality in digital agriculture	EU
52	Analysis of the gender-blind perspectives in the context of digitalisation and Work	Germany
53	Designing gender-inclusive digital solutions for agricultural development	Global
58	Building a bridge between debates on welfare and gender on one hand, and the social impact of algorithms on the other	Global
*Exclusion from digital governance and institutional leadership*		
59	Identification of actors who drive digital revolution in agriculture	Global
66	Identification of how data-driven agricultural business models lock farm data into machines and devices, reducing downstream agricultural services market competitiveness	EU/USA

### Barriers at the level of digitisation of business processes

At this level, gender inequality is reproduced through constraints on resources, including time, knowledge, technology, and operational autonomy.

***Socio-cultural norms.*** Socio-cultural norms continue to construct farming as a masculine domain, influencing both representation and material access to resources and authority in many rural European contexts. For example, women account for only 13% of farm holders in Ireland, despite substantial involvement in farm management. ^19^ Cultural expectations about gender reduce women’s likelihood of holding legal title, reducing eligibility for formal credit, subsidies, and many innovation programmes, which depend on documented asset ownership.

The effects of these norms extend beyond ownership. Women in agriculture frequently shoulder a double burden of unpaid or unrecognised farm work together with household and caregiving responsibilities, which constrains their time, mobility, and capacity to engage with market and technical opportunities. ^6^ Life stories from Greece show that, even where women’s formal participation in farming has increased, hierarchical rural structures and gendered expectations persist. The women enter farming through non-traditional pathways and revise their self-identity, yet remain subject to restrictions on the tasks deemed appropriate, the times and places they can work, and the forms of expertise that confer legitimate authority. ^20^

In addition, institutional and service delivery practices reinforce these dynamics. Extension and capacity-building activities are often scheduled or delivered in formats that are inaccessible to women with family responsibilities, and the limited presence of female extension agents reduces women’s uptake of technical support and market information, thereby constraining innovation and productivity. ^21^ Early socialisation and gender-biased pedagogies, together with a lack of visible female role models in agricultural and STEM fields, further discourage young women’s aspirations and confidence. For instance, research from Moldova reports attrition among girls who initially expressed interest in information and communication technology (ICT) careers, citing beliefs that such careers are unsuitable for women. ^22^ These educational and cultural processes operate as upstream drivers that shape occupational choices and long-term access to technical skills and networks.

Although younger generations of both men and women appear less tolerant of overt discrimination, ^21^ cultural norms around farming and gender remain adaptable. They can absorb increased female labour and participation without altering the underlying distribution of assets and authority. This passive feminisation allows visible gains in participation to coexist with persistent material and institutional inequalities, so that apparent increases in women’s presence on farms may obscure rather than resolve deeper structural barriers. ^20^

***Time and labour burden.*** Women comprise approximately 35% of the agricultural workforce, yet they are often employed in unpaid, part-time, or family-worker roles that are not captured by official statistics. ^6^ Domestic and care responsibilities assigned to women reduce the time available for paid employment, skills development, training participation, and decision-making involvement. This results in time poverty, which constrains women’s agency. ^23^ Over time, these constraints lead to diminished income security, restricted access to social protection, and lower pension accumulation, thereby reinforcing long-term economic vulnerability.

At the policy level, these inequalities are perpetuated by the sectoral framing of agriculture. As Shortall and Marangudakis ^24^ argue that EU agricultural policy primarily defines agriculture in terms of land, production, and subsidy distribution, rather than as an occupation shaped by gendered divisions of work. As a result, instruments such as the Common Agricultural Policy (CAP) allocate support based on land ownership and farm size, criteria that systematically favour men, who are more likely to control larger and more profitable holdings. In contrast, women are disproportionately represented in smaller-scale or subsistence-oriented farms, many of which do not qualify for substantial support. This structural disconnect between labour contribution and resource allocation reinforces gendered inequalities in income and investment capacity.

Gendered expectations influence both the types of tasks women perform and the allocation of time within households. Comparative time-use studies across Europe indicate that countries with more traditional gender norms display greater disparities in both paid and unpaid work. In contrast, more egalitarian welfare regimes and family policies are associated with narrower gaps, suggesting that labour inequalities are equally shaped by institutional context rather than being culturally fixed. ^25^

Cross-national evidence supports this structural perspective. For example, Román and Gracia ^26^ demonstrate that gender gaps in unpaid care work and paid employment follow a consistent life-course trajectory, widening during adolescence, peaking in early adulthood, and narrowing later in life. The magnitude of these gaps, however, varies considerably across countries, with more pronounced inequalities in Southern Europe compared to more egalitarian welfare contexts such as Sweden. Similarly, Anxo et al. ^27^ show that variations in family policy regimes directly influence the distribution of paid and unpaid labour, highlighting the significance of institutional design in either perpetuating or mitigating gendered time inequalities.

Nevertheless, increased female labour force participation has not necessarily resulted in economic empowerment. Instead, it is often associated with the growth of part-time, precarious, and low-paid employment. Filandri and Struffolino ^28^ demonstrate that high female employment rates do not inherently reduce poverty when job quality is poor and social protection is inadequate. In these contexts, labour market inclusion may coexist with, or even exacerbate, economic insecurity. This challenges policy assumptions that participation alone is sufficient for empowerment and underscores the importance of how labour is structured, valued, and compensated.

***Limited access to information.*** Access to information is a critical resource in contemporary agricultural systems, especially amid increasing digitalisation and knowledge-intensive production. Nevertheless, women’s access to extension services, advisory platforms, and professional networks remains constrained. ^2^ This exclusion arises not primarily from the availability of services, but from the structural orientation and intended beneficiaries of agricultural knowledge systems. These systems frequently presume a default user who is male, formally recognised as the farm holder, and regarded as the primary decision-maker.

This bias is further reinforced within family farming structures, where women are frequently assigned secondary or supportive roles. In regions such as Italy, the gendered division of labour reduces women’s visibility as economic actors and limits their direct engagement with extension services, training programmes, and professional networks. ^29^ Because access to advisory systems is often mediated through recognised farm ownership or leadership roles, women’s exclusion from these positions results in restricted access to information. Thus, informational inequality is systematically produced through the interaction of social norms and institutional design. ^6^

Limited access to information constrains the adoption of new technologies, reduces opportunities for business diversification, and weakens engagement with markets and innovation systems. ^30^ These limitations negatively affect economic performance and reinforce women’s concentration in lower-value segments of agricultural production. A study by Amato et al. ^29^ demonstrates that disparities in access to key resources, particularly information, are a significant determinant of lower entrepreneurial income among women farm holders, with the gap widening at higher income levels. These findings indicate that informational constraints do not merely limit entry into agricultural markets but also restrict upward mobility within them.

***Low digital literacy and confidence.*** Digital literacy is an enabling resource in data-driven agricultural systems, yet gendered disparities in skills and confidence continue to limit women’s meaningful engagement with digital technologies. While aggregate indicators suggest a narrowing gender gap in basic digital skills among younger cohorts in the EU, these figures mask persistent, structurally embedded exclusion that disproportionately affects older rural women, particularly those over 45 years working in agriculture. ^31^ This divergence underscores a limitation in current policy approaches, which often prioritise entry-level digital inclusion but overlook the uneven distribution of capabilities necessary for effective and autonomous technology use.

Literacy gaps stem from cumulative structural inequalities. Lower levels of formal education among older rural women, especially in Southern and Eastern Europe constrain their capacity to acquire digital skills. ^32^ According to the United Nations Educational, Scientific and Cultural Organisation (UNESCO), ^33^ women with only primary education are less likely to use digital tools, while those with secondary education are up to six times more likely to participate in digital environments. However, education alone does not fully account for the persistence of the digital literacy gap. Rural infrastructural deficits, such as limited access to training, inconsistent broadband quality, and demographic pressures including ageing populations and youth outmigration, exacerbate educational shortfalls and contribute to long-term exclusion from digital opportunities. ^33^

Moreover, digital inequality is influenced not only by deficits in technical skills but also by variations in confidence and perceived competence. Socio-cultural norms that associate technology with masculinity undermine women’s self-efficacy, particularly regarding advanced applications such as financial management platforms or farm decision-support systems, leading to an aversion to exploring advanced digital features. ^34^ Findings from the Horizon 2020 DESIRA project show that while rural women frequently use digital tools for routine or small-scale farming tasks, they are more hesitant to adopt advanced technologies. This pattern demonstrates that digital exclusion is stratified rather than absolute, with women more likely to engage in less advanced uses while remaining excluded from domains that provide greater strategic and economic benefits. ^35^ Additionally, digital interfaces that rely on technical language, assume prior expertise, or are available only in dominant national languages create further barriers for users with varying literacy levels or linguistic backgrounds. As a result, women are less likely than men to use digital technologies for fundamental tasks, with estimates indicating a gap of approximately 25% in functional usage. ^33^ The distinction between access and effective use is important, as nominal access to digital tools does not lead to empowerment if users lack the confidence and capability to use them strategically. This limitation constrains the ability to translate digital access into agency and economic outcomes, thereby perpetuating existing inequalities.

Initiatives such as the Agri-Digital Bildung Living Lab in Germany demonstrate potential strategies for addressing digital literacy gaps by integrating experiential learning, vocational training, and on-farm experimentation. Embedding digital skill development within real production contexts and fostering collaboration among students, farmers, educators, and researchers can help bridge the gap between access and effective use. However, the effectiveness of these initiatives depends on broader structural conditions, including sustained access to training, institutional support, and the redistribution of time and resources necessary for women’s participation. ^36^

***Inadequate device access and connectivity.*** Policy discourse frequently frames access to digital devices and connectivity as a technical matter of infrastructure provision. However, observed inequalities in connectivity stem not only from coverage gaps but also from unequal ownership, control, and use of digital resources. ^37^ Gallistl *et al*. ^38^ in a study with older adults in Austria, challenge this reductionist perspective by demonstrating that digital non-use is a negotiated practice shaped by social, cultural, and generational expectations. These practices transcend a simple binary between use and non-use, encompassing nuanced forms of engagement such as relying on others for digital tasks, sharing devices within family or community networks, or selectively engaging with certain technologies while avoiding others. However, current digital interventions typically prioritise connectivity metrics and overlook the lived experiences of exclusion that extend beyond coverage maps and broadband speeds. Consequently, initiatives that focus exclusively on rural broadband expansion risk reinforcing, rather than mitigating digital marginalisation.

Power dynamics within households and communities further shape access to digital technologies. Perifanou and Economides ^39^ demonstrate that persistent gender inequalities in rural contexts shape the allocation of devices and digital resources. When technology and digital skills are viewed as scarce, they are often allocated preferentially to men or younger family members. As a result, the prevailing assumption that rural women lack digital skills often reflects structural exclusion rather than individual capability. Addressing this issue requires more than providing training opportunities but also confronting patriarchal norms that dictate who may access, own, or control digital tools. Elena-Bucea *et al*. ^40^ similarly contend that the affordability barrier extends beyond the initial cost of devices or internet subscriptions. Ongoing income disparities and precarious rural economies mean that maintaining connectivity involves recurring expenses, which can be especially burdensome for single-income or female-headed households. Policies focused solely on one-time device provision, therefore fail to account for the sustained financial commitments necessary for digital access. Furthermore, the exclusion of rural women from digital resources has broader implications for families and communities. Limited access restricts participation in agricultural innovation, e-government services, and social networks, thereby perpetuating cycles of marginalisation and economic disadvantage. These dynamics underscore the limitations of market-driven digital inclusion strategies that narrowly emphasise infrastructure and consumer access.

Van Deursen and Van Dijk ^37^ highlight the limitations of equating internet connectivity with digital empowerment. As basic access becomes more widespread, inequalities increasingly manifest in material differences such as the number, quality, and diversity of devices available, as well as the ongoing costs of maintaining them. In rural Europe, this is evident in the rise of mobile-only households, many of which are led by women. While smartphones enable basic communication, they are often insufficient for more complex digital activities. Tasks like managing agricultural subsidy applications, accessing detailed farming data, or participating in online education typically require larger screens, stable connections, and more advanced devices. As a result, dependence on mobile devices can limit users to passive or limited forms of online engagement. When digital interventions fail to address these material disparities, they risk presenting a superficial appearance of inclusion while deeper forms of exclusion persist.

### Barriers at the level of digitalisation of ecosystems

At the level of digital ecosystems, gender inequality is most visible in constraints on agency, understood as women’s capacity to make strategic decisions, participate in markets, and exercise voice within digitally mediated agricultural networks. These barriers shape whether access can be translated into meaningful choice.

***Financial exclusion.*** A central driver of financial exclusion is the persistent gender disparity in asset ownership. Women generally possess fewer productive assets than men, particularly agricultural land, which serves as the primary collateral in formal credit markets. ^19^ Since financial institutions predominantly require land titles and other high-value assets to secure loans, women’s lower rates of ownership directly reduce their credit eligibility. This limited access to credit constrains women’s ability to invest, expand production, and generate stable income, thereby perpetuating a weaker asset base and reinforcing cycles of disadvantage. Initiatives aimed at increasing female land registration have yielded ambiguous outcomes that do not correspond with headline statistics. While official data report a rise in female farm holders, these numbers do not necessarily indicate changes in control or decision-making authority. ^5^

Tsiaousi and Partalidou ^41^ show that women formally registered as farm heads often lack primary authority over land management. Their designation often enables households to access subsidies or maximise policy benefits. This phenomenon results in what is termed statistical inclusion, where women appear integrated into formal agricultural and financial systems, yet strategic control over resources remains with men. ^1^ ^,^ ^42^ These improvements in registration-based metrics may lead policymakers to interpret these indicators as evidence of transformation, even as deeper structural inequalities persist. As Maftei ^43^ argues that without addressing normative constraints, increases in female registration or participation are likely to remain largely symbolic. Legal reform alone is insufficient to alter authority structures if prevailing gender norms continue to determine legitimate control over resources.

***Market access constraints.*** Market constraints arise from the disproportionate concentration of women in small-scale or subsistence farming. ^20^ Smaller landholdings typically yield lower output volumes, restricting the ability to generate consistent marketable surpluses. Participation in wholesale, export, and other high-value markets generally requires scale, regular supply, and logistical capacity. Consequently, women farmers are frequently restricted to local or low-margin sales channels, which perpetuates income disparities.

In addition, women’s roles have historically been limited to local markets or household food production, whereas men have dominated the more profitable domains of external trade and large-scale market transactions. ^44^ These gendered expectations are reinforced by the unequal distribution of unpaid care work, exemplified in Lithuania, where women predominantly bear the responsibilities of childcare and domestic labour. This dual burden restricts women’s time, mobility, and ability to develop business networks or access distant markets, intensifying barriers to market participation. ^45^

Women are also underrepresented in producer organisations and cooperatives, which are essential for providing market intelligence, facilitating access to new technologies, enabling collective branding, and strengthening price negotiations. ^18^ The absence of collective bargaining power and limited access to market information perpetuate a cycle of low returns and weak negotiating positions. Additionally, gender-based biases among buyers and intermediaries frequently result in women’s products being undervalued, compelling them to accept lower farm-gate prices. ^21^

While short supply chains, such as farmers’ markets and direct sales can offer women opportunities to bypass traditional barriers and capture higher margins, they also introduce some challenges. According to Dupé *et al*., ^46^ these models require farmers to take on a wide range of responsibilities, including direct marketing, customer communication, order processing, logistics coordination, compliance with certification requirements, financial administration, and participation in collective governance. These tasks increase transaction frequency and demand sustained relational and administrative engagement, often extending beyond standard working hours. For women farmers who disproportionately shoulder unpaid care and household responsibilities, such demands may constitute a structural barrier. The Cloughjordan Living Lab in Ireland exemplifies both the challenges and transformative potential of short supply chains. Its user-owned digital food hub allows producers to upload products to an online marketplace, manage orders, and coordinate deliveries to a shared collection point where payments are centrally processed and redistributed. ^36^ While this system reduces intermediaries and enhances local market access, it also requires continuous digital engagement and logistical coordination. Because the platform was co-developed with participating farmers, it aims to streamline administrative tasks and distribute organisational responsibilities collectively, thereby mitigating rather than exacerbating the labour burdens typically associated with short supply chains.

Moreover, these gendered market access constraints extend beyond the EU context and reflect broader global patterns. In global value chains, as Bamber and Staritz ^47^ demonstrate, increased participation by women does not necessarily result in equality. Gendered segmentation persists, with women concentrated in lower-value, lower-paid roles and continuing to face challenges in accessing finance, information, and productive resources. Therefore, economic upgrading in value chains does not automatically lead to social upgrading or a reduction in gender disparities.

***Safety and trust issues.*** While digitalisation presents great opportunities for rural development and agricultural innovation, these advancements are accompanied by safety and trust concerns that disproportionately impact women and girls. These issues are evident in the widespread occurrence of online violence, harassment, and cyberstalking. Such abuse leads to psychological distress, reputational harm, and fear of exposure, which collectively discourage women from fully engaging in digital agricultural platforms, online markets, and professional networks. ^48^ This limitation directly restricts their access to information, funding, training, and market connections, all of which are increasingly facilitated through digital channels. ^49^ A report by the European Commission ^50^ indicates that one in ten women in the EU has experienced some form of cyber violence since the age of 15. The actual prevalence is likely higher due to stigma, the normalisation of online abuse, and widespread underreporting. Underreporting perpetuates the cycle of harm by reducing the visibility of the issue, which delays policy responses and weakens institutional accountability.

Although the EU has made progress, including the adoption of the 2024 Directive to combat violence against women by criminalising various forms of cyber violence, significant gaps remain in the recognition and enforcement of these standards at the national level. ^51^ Many Member States do not provide comprehensive definitions of gender-based digital violence, often resorting to gender-neutral language in legislation that fails to address the disproportionate targeting of women and girls. This leads to inconsistent protections, inadequate enforcement, and insufficient intersectional awareness, particularly disadvantaging rural women who may also experience marginalisation based on age, ethnicity, or immigrant status.

### Barriers at the level of digital transformation of socio-ecological systems

At this level, barriers are embedded in data systems, governance structures, and institutional priorities, shaping who is visible, who participates, and who benefits from digital transformation of agriculture.

***Gender-blind design of technologies.*** The design and implementation of agricultural technologies have historically presumed user neutrality, implicitly treating male experiences, work patterns, and physical characteristics as the normative standard. ^52^ This presumption has led to the exclusion and marginalisation of women at multiple levels of agricultural participation. Ergonomic biases are particularly evident in the development of farming equipment, as tools and machinery are frequently designed based on average male body dimensions and strength profiles. ^53^ As a result, women often face ergonomic mismatches that render certain tasks more physically demanding or hazardous. These mismatches can reduce efficiency, increase fatigue, elevate injury risks, and reduce adoption of technologies, thereby constraining productivity and perpetuating gendered divisions of labour. ^54^

Moreover, even when gender issues are acknowledged, insufficient data impedes the development of targeted interventions, training materials, or support services that address women’s specific needs and contexts. ^55^ ^,^ ^56^ Women’s work, which is disproportionately concentrated in part-time, seasonal, and unpaid labour, is often underrepresented or omitted from official data collection frameworks. ^57^ Additionally, self-reporting practices often reflect internalised social norms, with women more likely to identify as helpers rather than primary farmers. These dynamics not only represent descriptive limitations but also actively generate a distorted portrayal of the agricultural workforce, positioning male, full-time farm holders as the implicit reference category. ^1^ As a result, women’s contributions are not merely overlooked but are structurally misrecognised within the data systems that inform policy and resource allocation. ^58^

EU policymaking, including the design and implementation of the Common Agricultural Policy (CAP) depends on statistical indicators to determine eligibility, allocate subsidies, and assess performance. When these indicators are derived from gender-blind or incomplete datasets, they reinforce a narrow model of farming that reflects male-dominated ownership and production structures. ^1^ ^,^ ^5^ Consequently, support schemes, advisory services, and research priorities are not only misaligned with women’s experiences and needs but are also structurally predisposed to exclude them. In this way, gender inequality becomes embedded within the metrics and categories that shape agricultural systems.

***Data disempowerment.*** As farm-level data becomes increasingly central to innovation, decision-making, and market coordination, the governance of data rights becomes critical. ^48^ ^,^ ^57^ ^,^ ^59^ However, existing frameworks around data ownership, consent, and usage fail to account for intra-household power asymmetries and gendered differences in digital literacy and decision-making power. ^55^ ^,^ ^60^ Women may formally consent to data sharing while lacking control over how that data is used, monetised, or integrated into platform-based systems. ^61^ As a result, women remain underrepresented in datasets that shape policy and innovation, while they are simultaneously incorporated into data extraction processes under unequal conditions. As Van der Burg *et al*. ^62^ suggest, such arrangements risk positioning women not as beneficiaries of digital transformation, but as peripheral data subjects within systems that generate value elsewhere.

Although gender mainstreaming has been formally incorporated into EU policy frameworks, its implementation often remains procedural rather than transformative. ^4^ Instruments such as gender impact assessments, training programmes, and gender budgeting are typically added to existing systems without modifying the underlying logic of eligibility, measurement, or accountability. ^63^ This approach leads to institutional inertia, where the appearance of inclusion persists alongside structurally biased policy architectures. Furthermore, the lack of robust monitoring mechanisms limits the ability to identify or address these systemic distortions. ^1^

***Exclusion from digital governance and institutional leadership.*** Digitalisation in agriculture constitutes a politically mediated transformation, rather than merely a process of technological adoption. ^64^ It is shaped by coalitions of state agencies, producer organisations, corporate actors, and expert communities that collectively define strategic priorities, allocate resources, and design regulatory frameworks. ^59^ Within these arenas, women and gender-focused organisations remain underrepresented, concentrating agenda-setting power among actors whose perspectives are often primarily oriented toward productivity and market competitiveness. ^65^ This imbalance helps explain why gender equality objectives in EU policy frameworks frequently remain peripheral to dominant priorities such as efficiency, innovation, and trade. ^4^

Furthermore, the governance of digital agriculture, including data standards, interoperability frameworks, innovation funding, and the regulation of platforms and algorithms, is likewise negotiated in spaces where women are underrepresented and rarely occupy senior decision-making roles. ^66^ As a result, implicit assumptions about who qualifies as a farmer, innovator, or legitimate data subject risk becoming embedded in the design of digital systems and policies.

Similar dynamics are evident at the national level, where ministries, advisory bodies, and industry-led initiatives responsible for digital strategies in agriculture and rural development often reproduce existing gender imbalances. Even where women’s organisations are formally included, their influence may remain limited due to restricted access to key decision-making spaces and insufficient financial or technical resources to engage effectively in complex debates on data governance, artificial intelligence, and platform regulation. ^2^

## Artificial intelligence and the reconfiguration of gendered barriers

Recent advances in artificial intelligence, particularly Large Language Models (LLMs), are increasingly positioned as gender-transformative tools capable of reshaping agricultural systems. ^67^ Multimodal functionalities incorporating voice, text, and image interaction reduce traditional literacy and language barriers. However, research by Aneja et al. ^68^ highlights that because many LLMs are trained on formal scientific journals and administrative datasets, they often produce outputs laden with formal jargon that fail to recognise the non-standard terms used by women farmers for cropping practices. This linguistic mismatch can reinforce a digital divide even as it attempts to bridge it. ^69^

Effective engagement with AI necessitates not only digital literacy but also a design-by-inclusion approach grounded in gender equality, diversity, and inclusion principles. ^70^ Reviews of AI bias in agriculture highlight that, without such intentional design, AI systems risk perpetuating algorithmic bias, as demonstrated when speech recognition programs or search results favour the demographics most represented in their training data ^71^ As a result, participatory engagement is increasingly recognised as essential for enhancing the relevance and usability of AI tools among marginalised groups. ^70^

Additionally, although AI-enabled automation has the potential to alleviate time poverty by streamlining administrative and labour-intensive tasks, its effectiveness is significantly influenced by prevailing household norms. A study by Di and Li ^72^ indicates that digital tools could theoretically reduce housework by approximately 30%; however, urban-rural disparities in adoption restrict these benefits for rural women. Moreover, the time saved through mechanisation or automation is often reallocated to additional unpaid care, domestic chores, or child-rearing activities, rather than being redirected toward income-generating or decision-making roles. ^73^

At the decision-making level, AI-based advisory tools offer private, on-demand information that can circumvent male-dominated extension networks. Reeves *et al*. ^69^ report that machine-supported decision-making increases female participation in agricultural training by optimising for gender-specific preferences, such as session timing and location. However, intra-household hierarchies continue to determine ownership of digital credentials and devices. ^64^ In predominantly patriarchal heritage contexts, men frequently control access to technology or interpret AI-generated guidance, so formal access does not necessarily translate into decision-making autonomy.

AI technologies also shape epistemic authority by prioritising algorithmically generated metrics over experiential and relational knowledge. When AI systems are trained on formal, biased datasets, they risk marginalising the indigenous and context-specific expertise of women who have historically been excluded from formal agricultural institutions. ^74^ Prioritising AI-derived outputs may further diminish recognition of women’s expertise unless gender-responsive data practices and participatory design are integrated from the outset. ^68^ Excluding diverse knowledge forms not only limits system relevance but also reinforces cultural hierarchies that marginalise women’s contributions to agricultural innovation. ^74^

AI’s dependence on large-scale datasets requires robust, gender-responsive data governance to prevent structural disempowerment. When datasets are gender-blind or reflect historical biases, they institutionalise inequality by rendering women invisible as economic actors while exposing them as data subjects. ^71^ Addressing these feedback loops necessitates frameworks that prioritise data rights, transparency, and the deliberate inclusion of women’s labour and economic contributions in AI training processes to ensure that digital transformation does not exclude marginalised farmers. ^53^

## Harnessing the potential of digital technologies in levelling gender gaps

Harnessing digitalisation to tackle deep-rooted gender barriers in EU agriculture offers exciting possibilities but also brings significant challenges. While digital transformation holds the potential to disrupt traditional power structures, its effectiveness depends on how interventions are designed, implemented, and situated within broader socio-cultural and institutional contexts. If digital interventions are not carefully planned with an awareness of gender issues and if they lack strong policy support, there is a real risk they could actually make inequalities worse, even while appearing to make progress. ^1^ ^,^ ^6^

One step toward advancing gender equality lies in increasing women’s visibility and agency through digital platforms. Online forums, social media campaigns, and e-learning initiatives not only facilitate access to information and resources but also serve as powerful platforms for challenging the idea that farmers are typically male. ^19^ ^,^ ^45^ However, these digital platforms can only truly make a difference if they reach women who are often left behind, especially those who are not confident with technology or who are busy with household responsibilities. Flexible, women-focused online services, and peer networks can help include more women, ^21^ but their impact is still limited by ongoing digital gaps. This is especially true for older women in rural areas. ^38^ Moreover, the tendency of digital campaigns to attract primarily those already inclined towards innovation risks deepening, rather than bridging, gender gaps if complementary offline strategies are neglected.

The implementation of labour-saving digital tools and remote advisory services represents a promising approach for alleviating the time poverty that characterises women’s agricultural and domestic workloads. ^23^ ^,^ ^25^ By making tasks more efficient and allowing women to connect with markets from a distance, these technologies have the potential to boost women’s independence and earning power. However, such benefits are dependent on the quality of the technology, the relevance of content, and the ease of device access and connectivity. For instance, one-off device provision schemes are unlikely to succeed unless they are accompanied by ongoing support, affordable data plans, and a focus on the family dynamics that influence who gets to use technology and how. ^38^ ^,^ ^40^

Addressing systemic barriers at the digital ecosystem level holds significant potential to advance women’s financial inclusion and market participation by improving their access to credit, insurance, and e-commerce. ^5^ However, persistent challenges such as collateral requirements, land ownership constraints, and hidden algorithmic biases can still pose significant obstacles. Therefore, achieving genuine inclusion requires combining digital finance solutions with broader structural reforms. These reforms should include transparent and participatory algorithm design and the systematic integration of gender-disaggregated data to prevent digital tools from replicating the very biases they aim to overcome. ^58^ Similarly, while e-commerce platforms and digital cooperatives can enhance women’s market access and collective bargaining power, they too are not complete solutions on their own.

Additionally, building safe and trusted digital ecosystems is paramount to empowering rural women, enabling their full participation in agricultural innovation and rural leadership. ^48^ ^,^ ^50^ This requires a dual approach: first, the consistent and robust enforcement of EU directives that criminalise gender-based cyber violence across all Member States. ^51^ Second, it necessitates the proactive design of inclusive online platforms featuring strong moderation, clear safety protocols, and community guidelines developed with an intersectional lens. ^49^ By combining rigorous legal protection with intentionally safe digital spaces, online harassment can be effectively mitigated to ensure that digital platforms become engines of opportunity for rural women.

Furthermore, to achieve change at the level of socio-ecological system, data governance frameworks should be redesigned to guarantee that women’s contributions are recognised, their data rights are upheld, and their agency is honoured. Participatory research and inclusive policymaking are essential for the co-creation of digital agricultural solutions that reflect and respond to the real needs of diverse women. ^57^

## Policy recommendations

Building upon the multi-level CODECS framework and the evidence presented, this section proposes policy recommendations to advance gender equality in EU agriculture and rural areas through equitable digitalisation.

### Enhancing gender equality at the business process level

***Integrate gender sensitivity into digital literacy and capacity building.*** Policymakers at the national and EU levels should develop and include gender-specific digital skilling programmes, tailored to the needs of rural women across age groups and educational levels. The initiatives should prioritise older women and those with little formal education, using context-appropriate training formats, local language materials, and flexible delivery methods. ^75^ Collaboration with local women’s organisations, agricultural extension services, and educational institutions is essential for effective outreach. In addition, the involvement of mentors and female role models can promote trust and acceptance in rural communities.

***Facilitate equitable access to digital devices and connectivity.*** Targeted subsidies and financial support for digital device acquisition and broadband access should be made available to women in rural and agricultural settings, especially female-headed households. Public-private partnerships should be incentivised to develop and scale mobile-first solutions (e.g., SMS, voice-based services) that are suitable for basic-level devices and varying literacy levels. Policy makers should ensure that digital inclusion efforts go beyond basic connectivity to consider sustainable affordability and usability. ^57^

***Formal recognition of women’s labour.*** Statistical and administrative protocols should be reformed to recognise both paid and unpaid labour contributions by women in agricultural systems. Mandating the use of digital farm management platforms that record women’s roles and outputs can facilitate access to credit, social protection, and agricultural subsidies. It is also essential that all agricultural data collections produce and report sex-disaggregated statistics to enable evidence-based policy. ^1^

### Fostering gender equality within the digital ecosystems

***Promote gender-responsive financial innovation.*** Financial inclusion should be improved by redesigning digital credit, insurance, and payment platforms that take into account the specific constraints faced by rural women, such as limited collateral and informal credit histories. Regulators should require a gender review of algorithms used in digital finance, to ensure that automated systems do not reproduce biases. Collaboration with women’s cooperatives and rural financial institutions should be prioritised in the co-development of tailored digital financial products. ^43^

***Strengthen market access and entrepreneurship.*** Support for women’s participation in digital value chains needs to be scaled up by providing funding, technical assistance, and capacity-building for women-led cooperatives, e-commerce initiatives, and digital marketplaces. ^76^ Technology developers and agribusiness platforms should be required to consult and involve women users throughout product development and piloting to ensure usability and inclusivity. ^77^ Targeted support should be provided for digital competences, marketing skills, and collective bargaining to enable women to navigate and succeed in digital markets.

***Ensure safe, inclusive, and empowering digital spaces.*** Digital agricultural platforms need to enforce robust online safety standards, including proactive moderation, transparent reporting mechanisms and explicit anti-harassment policies. It is recommended to invest in the creation and support of women-only or women-led digital communities, including forums and mentorship networks, to create safe spaces for knowledge sharing, leadership and advocacy. ^78^

### Advancing structural transformation at the socio-ecological system level

***Institutionalise gender-responsive co-design and innovation.*** Participatory co-design approaches, such as those demonstrated in CODECS Living Labs, should be institutionalised as a standard practice in all publicly funded digital agricultural innovation projects. The active participation of women as co-creators and end-users is crucial for the relevance, acceptance, and impact of new technologies. ^79^ Concurrently, guidelines for gender-responsive digital innovation should be developed at both the national and EU-level, and compliance with these guidelines made a prerequisite for funding.

***Reform data governance for equity, agency, and accountability.*** Policymakers should mandate the systematic collection and publication of sex-disaggregated data and require regular gender impact assessments for all digital agricultural interventions. Additionally, data rights frameworks should empower women to control and benefit from agricultural data generated by and about them, with explicit consent protocols and transparency. Further, the establishment of independent monitoring bodies to oversee adherence to gender equity standards in digital agriculture is strongly recommended. ^1^

***Ensure policy coherence and long-term sustainability.*** Digital agriculture strategies should be aligned with existing frameworks for gender equality, rural development and the Sustainable Development Goals (SDGs). Moreso, cross-sectoral working groups, integrated funding streams and harmonised monitoring and evaluation mechanisms will be essential for effective and sustainable impact. ^56^ We further recommend that policies are regularly reviewed and updated based on empirical evidence and stakeholder feedback.

## Conclusion

Digitalisation in EU agriculture does not inherently drive gender transformation but rather reconfigures resources, capabilities, and control in ways that intersect with existing gender inequalities. Although digitalisation may expand access to information, markets, and innovation, these advantages are distributed unevenly and frequently do not result in significant increases in authority or economic autonomy. Consequently, digitalisation can perpetuate integrated forms of inequality, whereby women’s participation rises without parallel changes in power, recognition, or control over resources. This situation challenges policy approaches that emphasise access and inclusion but overlook the structural factors influencing outcomes. Addressing these issues requires strategies that go beyond access-based interventions to address underlying power asymmetries, such as implementing gender-responsive data infrastructures, ensuring accountability in digital governance, and enhancing women’s inclusion in decision-making processes.

Insights from the CODECS Living Labs indicate that more inclusive trajectories are possible through participatory and co-creative approaches. However, their impact remains contingent on whether such practices extend beyond localised initiatives and influence broader institutional frameworks. Ultimately, the results of digitalisation are determined not by the technology itself, but by the actors who shape it, and the manner in which its benefits and costs are distributed.

## Data Availability

No raw data associated with this article. All the materials used are either published peer reviewed articles, policy documents, technical reports or even Living Labs reports (these are published on the CODECS website). All of which have been cited.
