# Supplementary material for: Leveraging digital solutions to address gender inequalities in EU agriculture: a CODECS framework application
Source: Open Res Eur. 2026 Apr 11;5:322. Originally published 2025 Oct 17. [Version 2] doi: 10.12688/openreseurope.21445.2 (PMC13146467; doi:10.12688/openreseurope.21445.2)
Supplement: Peer review history

# Reviewer report: Reviewer response for version 2

**DOI:** 10.21956/openreseurope.25450.r72858

**Published:** 2026-05-21

**Authors:** Niyati Singaraju

**Affiliations:** 1 International Rice Research Institute, IRRI-India Office, Hyderabad, India

The article examines how digitalisation can be used to address gender inequalities in EU agriculture using CODECS framework.

 It looks at gender barriers across three levels - business processes, digital ecosystems and broader socio-ecological systems.  It argues that digital tools can support women in agriculture by improving access to information, markets, advisory services, and networks, but only when these tools are designed with gender and context in mind. The article is careful not to present digitalisation as automatically empowering. It also shows how digital tools can reproduce existing inequalities through gender-blind design, weak data governance, limited digital confidence, financial exclusion, and exclusion from decision-making spaces.

 This is a good and a relevant piece

 - CODECS levels are explained better now and the discussion around data disempowerment, gender-blind design, autonomy have been addressed in this revised version. The article is presented in a lucid manner and authors have included relevant literature on the topic. It is not trying to present new quantitative data, but to bring together literature, policy documents, and CODECS Living Lab insights through a conceptual framework. This works well for the purpose of the paper. The use of the CODECS framework gives the paper academic merit because it allows the authors to move beyond listing barriers and instead show how gender inequality appears at different levels of the digital agriculture system. The paper also has policy relevance because it connects the analysis to practical recommendations on training, co-design, data governance, and safe digital spaces. The authors also explain how the evidence was mapped across the three CODECS levels. This is sufficient for a qualitative conceptual/policy-oriented paper. I do not think this needs further major revision. If the authors want to improve it slightly, they could add a short sentence explaining whether any documents were excluded and why. But this is not essential for the scientific soundness of the paper. Since this is not a primary empirical data paper, full reproducibility in the statistical sense is not really applicable. The conclusions are supported by the analysis. The paper does not overclaim that digitalisation will solve gender inequality. Instead, it argues that digital tools can help only when supported by better governance, gender-responsive design, data accountability, and institutional change. This is well supported by the material presented. My main suggestion is to make the idea of digital agency a little clearer. The article already talks well about access, use, empowerment, data disempowerment, and governance. But it could say more directly that digital agency is not only about whether women and marginalised groups can access or use a digital tool. It is also about whether they have the knowledge, confidence, and power to decide how to use it, when to use it, and whether to use it at all. 

 This point is important because many digital inclusion discussions stop at access and use. But real agency also includes the power to not use a tool, especially when there are concerns around data privacy, consent, surveillance, ethics, platform dependence, or algorithmic bias. For many women and marginalised users, these parts of digital tools are not visible. They may not know what data is being collected, how it is being used, who benefits from it, or what risks are built into the design of the tool. 

 So, I would suggest adding a few lines in the discussion or policy recommendations to say that empowerment should include informed choice and not just adoption. Women and marginalised groups should have the capability to understand what digital processes mean, what choices they have, and what the consequences are of using or not using a tool. This would strengthen the article’s existing discussion on data governance, gender-blind design, and participatory co-design. 

 This is a minor but useful addition. It does not require restructuring the paper. I do not see any major point that must be addressed for the article to be scientifically sound. The article is already scientifically acceptable in my view. 

 The point on digital agency is a recommendation to make the argument stronger and more complete, not a major flaw. The authors could address it with a short addition in the discussion or policy recommendations. It is a good, timely, and useful article. I recommend approval.

Is the study design appropriate and does the work have academic merit?

Yes

Is the work clearly and accurately presented and does it cite the current literature?

Yes

If applicable, is the statistical analysis and its interpretation appropriate?

Not applicable

Are all the source data underlying the results available to ensure full reproducibility?

Yes

Are the conclusions drawn adequately supported by the results?

Yes

Are sufficient details of methods and analysis provided to allow replication by others?

Yes

Reviewer Expertise:

I work on gender and digital inclusion in agrifood systems

I confirm that I have read this submission and believe that I have an appropriate level of expertise to confirm that it is of an acceptable scientific standard.

# Reviewer report: Reviewer response for version 2

**DOI:** 10.21956/openreseurope.25450.r72179

**Published:** 2026-05-05

**Authors:** Valeria Naciti

**Affiliations:** 1 University of Messina, Messina, Italy

Thank you for the revisions. I am satisfied with the changes made to the manuscript and the author responses, and I have no further comments at this stage.

Is the study design appropriate and does the work have academic merit?

Yes

Is the work clearly and accurately presented and does it cite the current literature?

Partly

If applicable, is the statistical analysis and its interpretation appropriate?

Not applicable

Are all the source data underlying the results available to ensure full reproducibility?

No

Are the conclusions drawn adequately supported by the results?

Yes

Are sufficient details of methods and analysis provided to allow replication by others?

Yes

Reviewer Expertise:

My research focuses on gender budgeting, gender accounting, and sustainability reporting, with a particular interest in how these frameworks contribute to promoting gender equality and transparency in both the public and private sectors. I also study the gender pay gap, exploring its determinants, implications, and the role of accounting and reporting practices in addressing this issue.

I confirm that I have read this submission and believe that I have an appropriate level of expertise to confirm that it is of an acceptable scientific standard.

# Reviewer report: Reviewer response for version 2

**DOI:** 10.21956/openreseurope.25450.r72180

**Published:** 2026-04-22

**Authors:** Ata Can Bertay

**Affiliations:** 1 Sabanci Business School, Sabanci University, Istanbul, Turkey

The revisions to my earlier comments are satisfactory.

Is the study design appropriate and does the work have academic merit?

Yes

Is the work clearly and accurately presented and does it cite the current literature?

Yes

If applicable, is the statistical analysis and its interpretation appropriate?

Not applicable

Are all the source data underlying the results available to ensure full reproducibility?

Partly

Are the conclusions drawn adequately supported by the results?

Yes

Are sufficient details of methods and analysis provided to allow replication by others?

Partly

Reviewer Expertise:

Gender and economic growth, corporate governance, financial intermediation

I confirm that I have read this submission and believe that I have an appropriate level of expertise to confirm that it is of an acceptable scientific standard.

# Reviewer report: Reviewer response for version 1

**DOI:** 10.21956/openreseurope.23194.r62833

**Published:** 2026-01-09

**Authors:** Valeria Naciti

**Affiliations:** 1 University of Messina, Messina, Italy

Your paper offers a rich and timely examination of gender inequalities in the digital transformation of European agriculture. It brings together an impressive range of empirical evidence, detailed policy insights, and practical illustrations from the CODECS Living Labs. The manuscript’s central argument, that digitalisation can both challenge and reproduce structural gender inequalities, is persuasive and highly relevant to current debates on sustainable rural development and inclusive innovation. However, while the paper’s empirical depth is commendable, its theoretical framing could be strengthened. In its current form, theory functions primarily as background rather than as a driver of analysis. Strengthening the theoretical integration would not only give the paper greater analytical coherence but would also make its contribution to academic debate clearer and more distinctive.

 At present, the text gestures towards multiple theoretical traditions, gender and development, digital inclusion, socio-technical systems, and sustainability transitions, but it never fully commits to one or shows how these perspectives interconnect. The argument would benefit from a more explicit articulation of its conceptual backbone. For instance, the analysis could be anchored either in a gender-transformative framework that seeks to expose and alter structural power relations, or in a socio-technical transitions perspective that views digitalisation as a multi-level process of change shaped by institutions and culture. Clarifying this would help readers understand not only *what* the paper observes, but *why* those patterns matter theoretically.

 The concept of empowerment also deserves deeper theoretical treatment. While empowerment is invoked throughout the paper, in relation to time, agency, and participation , it remains implicit rather than systematically defined. Incorporating a framework understanding of empowerment as a process of gaining the ability to make strategic life choices would offer conceptual clarity. It would also align neatly with the structure of your analysis: digitalisation can expand resources, enhance agency, and transform institutional structures. Framing the argument in this tri-dimensional way would bring coherence to the empirical sections and better connect them to broader debates in gender and development studies.

Is the study design appropriate and does the work have academic merit?

Yes

Is the work clearly and accurately presented and does it cite the current literature?

Partly

If applicable, is the statistical analysis and its interpretation appropriate?

Not applicable

Are all the source data underlying the results available to ensure full reproducibility?

No

Are the conclusions drawn adequately supported by the results?

Yes

Are sufficient details of methods and analysis provided to allow replication by others?

Yes

Reviewer Expertise:

My research focuses on gender budgeting, gender accounting, and sustainability reporting, with a particular interest in how these frameworks contribute to promoting gender equality and transparency in both the public and private sectors. I also study the gender pay gap, exploring its determinants, implications, and the role of accounting and reporting practices in addressing this issue.

I confirm that I have read this submission and believe that I have an appropriate level of expertise to confirm that it is of an acceptable scientific standard, however I have significant reservations, as outlined above.

## References

[1] : Gender diversity and performance in family small-to-medium business: Mapping and clustering bibliometric networks. *https://piccolaimpresa.org/article/view/1234 (replace with actual link if available)* .2021;

[2] : Gender in agriculture and food systems. *https://www.sciencedirect.com/book/handbook-of-agricultural-economics (replace with actual link)* .2021;

[3] : Factors influencing gender and youth integration in agricultural research and innovation in Africa. *https://link.springer.com/article/10.1186/s43170-024-00012-3* .2024;

# Response

**Published:** 2026-03-24

**Authors:** Barbra Comfort Akello

**Affiliations:** Agricultural, Food and Agro-Environmental Sciences, Not Applicable, Pisa, Italy

We thank the reviewer for her insightful and constructive comments, which have improved the quality of this manuscript.

**Reviewer’s comment**

 However, while the paper’s empirical depth is commendable, its theoretical framing could be strengthened. In its current form, theory functions primarily as background rather than as a driver of analysis. Strengthening the theoretical integration would not only give the paper greater analytical coherence but would also make its contribution to academic debate clearer and more distinctive.

 At present, the text gestures towards multiple theoretical traditions, gender and development, digital inclusion, socio-technical systems, and sustainability transitions, but it never fully commits to one or shows how these perspectives interconnect. The argument would benefit from a more explicit articulation of its conceptual backbone. For instance, the analysis could be anchored either in a gender-transformative framework that seeks to expose and alter structural power relations, or in a socio-technical transitions perspective that views digitalisation as a multi-level process of change shaped by institutions and culture. Clarifying this would help readers understand not only *what* the paper observes, but *why* those patterns matter theoretically.

 The concept of empowerment also deserves deeper theoretical treatment. While empowerment is invoked throughout the paper, in relation to time, agency, and participation, it remains implicit rather than systematically defined. Incorporating a framework understanding of empowerment as a process of gaining the ability to make strategic life choices would offer conceptual clarity. It would also align neatly with the structure of your analysis: digitalisation can expand resources, enhance agency, and transform institutional structures. Framing the argument in this tri-dimensional way would bring coherence to the empirical sections and better connect them to broader debates in gender and development studies. (Kabeer, 1999).

**Authors’ response**

 To strengthen the manuscript’s conceptual foundation, we adopt Kabeer’s (1999) tri-dimensional framework of empowerment (resources, agency, and achievements) as the basis for analysing how structural constraints affect individuals’ strategic life choices in agricultural and rural contexts. To apply this perspective, the study uses the CODECS multi-level framework, which organises the analysis across three levels: farm business processes, digital ecosystems, and broader socio-ecological systems. This approach enables a systematic examination of how empowerment dynamics are supported or limited across different scales of European agriculture.

 These changes are reflected in the “Introduction” ****section (blue text) and further detailed in the “Theoretical Framework” section (blue text) as outlined below.

*Introduction*

 Agriculture in the European Union (EU) remains characterised by deep-rooted gender inequalities, despite decades of political efforts to achieve equality ^1^ . Women continue to face significant barriers in accessing land, finance, education, technology, as well as meaningful decision-making power, leaving them at a disadvantage compared to men in almost all areas of agriculture and rural life ^2^ . Although gender equality is frequently cited in rural development objectives, many initiatives fail to address the underlying socio-cultural structures that reinforce this marginalisation ^3^ . As Shortall and Marangudakis ^4^ highlight, a fundamental structural imbalance of influence underpins the ineffectiveness of these measures. Dominant agricultural lobbying groups wield substantial influence over EU policy priorities, while gender-focused advocacy remains comparatively under-resourced and lacks robust legal and institutional backing. As a result, gender concerns are systematically sidelined in EU’s agricultural policy framework.

 The outcome of this neglect is adverse for women in rural areas, contributing to lower productivity, fewer entrepreneurial opportunities, limited economic independence, and diminished quality of life ^1^^, ^^4^ . This marginalisation is further evidenced by the reality that although women make up 35% of the agricultural labour force in the EU and contribute 31% of total working hours to the economy, their work continues to be undervalued, and their specific needs often remain unaddressed in policy and practice ^5^ . The persistence of these  gaps contrasts with the EU’s long-standing commitment to gender equality, enshrined in its treaties and reinforced by milestones such as the 1957 Treaty of Rome, the 1996 communication on gender mainstreaming, and the Gender Equality Strategy 2020–2025 ^6^ .

 From a gender and development perspective, this problem is best understood through the lens of empowerment, rather than representation or resource allocation alone ^7^. Empowerment is conceptualised here as a process through which individuals gain the ability to make strategic life choices in contexts where such ability has been systematically constrained ^8^. This process unfolds across interconnected dimensions, including access to resources, the exercise of agency, and the institutional conditions that enable or restrict the translation of resources and agency into sustained outcomes. Persistent gender inequalities in agriculture therefore reflect not only disparities in assets or opportunities, but enduring limitations on women’s capacity to influence decisions affecting their labour, livelihoods, and futures ^9^. 

 Digitalisation in agriculture is widely promoted in policy and industry circles as a strategic opportunity to redress the structural imbalances ^10^. Narratives associated with this so-called fourth agricultural revolution portray digital technologies such as artificial intelligence (AI), blockchain, and the Internet of Things (IoT) as neutral tools that can reduce labour burdens, improve access to information and markets, and enhance productivity ^11-13^. However, such techno-optimist accounts risk obscuring the social and institutional conditions under which digitalisation unfolds. Digital technologies do not operate in a vacuum as they are embedded in existing socio-technical systems and shaped by prevailing norms, power relations, and governance arrangements ^11^. Consequently, while digitalisation may expand women’s resources and agency, it may also reproduce or intensify gender inequalities by reinforcing existing patterns of exclusion in technologically mediated forms.

 Moreover, contemporary digital transformation differs fundamentally from earlier waves of technological change in agriculture, with distinct implications for gender relations. Historical mechanical innovations were largely characterised by a substitution effect, whereby machines replaced manual labour and directly reshaped rural labour markets. The post-war introduction of milking machines in Norway illustrates this dynamic clearly. Between 1930 and 1970, young rural women displaced from dairy farming were more likely to transition into better-paid, skilled employment, particularly in the public sector and to invest more in higher education ^14^. Although men were also affected by mechanisation, their displacement was less pronounced, and they did not experience comparable occupational upgrading. This pattern reflects a gender-biased technological change that contributed to the masculinisation of Norwegian agriculture, while, paradoxically narrowing long-term gender gaps in employment and education ^14^.

 By contrast, the digital technologies shaping present day European agriculture, such as AI, data platforms, and IoT, function less as direct substitutes for labour and more as cognitive and institutional layers that mediate information flows, decision-making processes, and governance arrangements ^12^. Rather than simply displacing work, digital systems influence who has access to data, whose knowledge is recognised, and who exercises authority within increasingly data-driven agricultural systems. As a result, digitalisation can generate divergent gendered outcomes. On the one hand, it may empower women by lowering physical barriers, expanding access to markets and knowledge, and enabling new forms of agency, including opportunities for innovation and change agency ^6^. On the other hand, it may reinforce exclusion through biased data infrastructures, unequal digital skills, and male-dominated governance structures, thereby intensifying a triple divide based on gender, digital access, and rural location ^11^.  Recognising this shift from labour substitution to cognitive mediation is essential for understanding why digital transformation in agriculture does not produce uniform gender effects.

 Current discussions on digital agriculture in the EU reflect this ambivalence ^10, 15, 16^. While a growing body of literature documents the economic and environmental benefits of digital technologies, far fewer studies systematically evaluate their implications for gender equality ^2^. Where gender is addressed, analyses often remain fragmented, focusing on isolated dimensions such as access to technology, labour participation, or entrepreneurship ^2, 4^. This has resulted in a lack of integrated explanations capable of clarifying how and under what conditions digitalisation of agriculture contributes to women’s empowerment, rather than merely describing its effects ^11^. The absence of a coherent framework capable of linking digital transformation to power, agency, and institutional change limits the analytical and policy relevance of the existing evidence.

 This study addresses this gap by employing an empowerment lens, operationalised through the framework developed in the Horizon project *Maximising the CO-benefits of agricultural Digitalisation through conducive digital ECoSystems* (CODECS). Instead of treating digitalisation as an isolated technological intervention, the analysis examines how empowerment processes develop within agricultural systems experiencing digital transformation. The CODECS framework conceptualises these systems across three interconnected levels: farm business processes, digital ecosystems, and broader socio-ecological systems ^17^. This multi-level perspective enables the identification of how opportunities for empowerment are facilitated or restricted at various scales, and how digital transformation interacts with existing governance structures, norms, and power relations in European agriculture.

 Guided by this integrated approach, we address two research questions:

 (1) What systemic gender-related barriers constrain women’s empowerment in EU agriculture?

 (2) Under what conditions can digital technologies and digital ecosystems contribute to gender-transformative change?

 By conceptualising digitalisation as a contested process of empowerment rather than a neutral technological shift, the study advances debates in gender and development, digital inclusion, and sustainable agricultural transitions. It contributes a theoretically grounded synthesis that highlights why digitalisation matters not only for productivity and innovation, but for the redistribution of power within European agriculture.

*Theoretical Framework*

 This study adopts an empowerment-based perspective to analyse how digitalisation reshapes gender relations in EU agriculture. It draws on the framework developed by Kabeer ^8^, which conceptualises empowerment as a process through which individuals gain the capacity to make strategic life choices in contexts where this ability has been systematically constrained. This approach is particularly suited to the analysis of gender inequality in agriculture, as it moves beyond descriptive accounts of participation to examine the underlying power relations that structure access to resources, decision-making, and outcomes.

 Kabeer’s framework distinguishes between three interrelated dimensions of resources, agency, and achievements. Resources encompass not only material assets such as land, capital, and technology, but also human and social resources, including skills, knowledge, and networks. Crucially, access to these resources is mediated by institutional rules and social norms, meaning that their distribution reflects existing power structures rather than neutral allocation mechanisms. Agency refers to the capacity to define goals and act upon them, extending beyond observable decision-making to include processes of negotiation, resistance, and the ability to envision alternative futures. Achievements represent the realised outcomes of these processes, indicating the extent to which individuals are able to translate resources and agency into meaningful improvements in their lives.

 While widely applied in development studies, the framework requires adaptation to capture the specific dynamics of digital transformation in advanced agricultural systems. Kabeer’s model does not explicitly account for the ways in which digital technologies mediate access to resources, reshape forms of agency, and influence institutional structures. Digitalisation introduces new forms of power, including control over data, algorithmic decision-making, and platform-based coordination, which cannot be fully understood through traditional resource-based analyses alone. As such, this study extends the empowerment framework by explicitly incorporating digital infrastructures as sites where power is produced and exercised.

 To operationalise this perspective, the study integrates Kabeer’s framework with the multi-level analytical structure developed in the CODECS project ^17^. This integration addresses a key limitation of empowerment-based approaches, which often focus on individual or household-level dynamics while under-theorising the broader systems within which these processes unfold. The CODECS framework enables the analysis of digitalisation as a system-level transformation, linking micro-level experiences of empowerment to meso and macro-level institutional dynamics.

 The analysis is structured across three interconnected levels.

 Level 1: Digitisation of business processes

 At this level, digitalisation affects the distribution of resources and the exercise of agency through access to technologies, digital skills, and decision-making authority. Gendered disparities at this level may manifest in unequal access to digital tools, limited participation in technology adoption decisions, and differential control over digitally mediated production processes. These dynamics directly influence empowerment outcomes, including productivity, income, and professional recognition.

 Level 2: Digitalisation of ecosystems

 At the ecosystem level, digitalisation shapes interactions among a broader set of actors, including advisory services, cooperatives, platforms, and markets. Here, power operates through network structures, institutional norms, and access to information flows. Participation in digital ecosystems can enable forms of collective agency and social capital accumulation but may also reinforce exclusion if governance structures remain male-dominated or if entry barriers such as digital literacy or platform access are unevenly distributed.

 Level 3: Socio-ecological systems transformation

 At this level, digitalisation is embedded within wider policy frameworks, regulatory regimes, and socio-economic institutions that shape the distribution of resources and opportunities. This includes control over data infrastructures, standard-setting processes, and the design of digital policies. These structures determine whether digital transformation reinforces existing gender inequalities or creates conditions for more transformative change by redistributing power and enabling new forms of participation.

 By linking empowerment processes across these three levels, the framework enables a more comprehensive analysis of how digitalisation reshapes gendered power relations in agriculture.

# Reviewer report: Reviewer response for version 1

**DOI:** 10.21956/openreseurope.23194.r66127

**Published:** 2026-01-06

**Authors:** Ata Can Bertay

**Affiliations:** 1 Sabanci Business School, Sabanci University, Istanbul, Turkey

This research article applies the CODECS conceptual framework to examine how digitalisation interacts with gender inequality in EU agriculture, integrating literature, policy analysis, and insights from CODECS Living Labs. It contributes by synthesising a fragmented literature and offering a structured analysis of complex, multi-level dynamics (in business processes, ecosystems, socio-ecological systems) to structure evidence on gendered digital divides in European agriculture.

 The topic is highly policy-relevant and suitable for Open Research Europe. However, the article would benefit from clearer methodological positioning, sharper conceptual definitions, and stronger analytical depth, including the causal mechanisms linking digitalisation to gender outcomes.

**Methodology and conceptual definitions:**

 -The use of the CODECS framework is appropriate and academically interesting. However, the paper lacks a clearly articulated study design. It is unclear: (1) how literature and policy documents were selected (search strategies or databases used), (2) how many Living Labs were included and on what basis, (3) how evidence was mapped onto the three CODECS levels (coding and categorisation procedures). The study currently reads more like a narrative synthesis than a systematic application of a framework. The authors may consider extending the methodological approach section to address this issue.

 -Related to the second issue mentioned, the article uses Living Lab cases, which are very insightful. Yet it would be great to provide more details, at least for one or two specific examples, describing how women were engaged, which digital tools were adapted or redesigned, and what changed in practice. This would considerably enhance the credibility of the empirical contribution.

 -Several concepts remain under-specified, for example, *“male farming practices”* in the discussion of design bias in agritech tools. To improve the article's clarity, the authors should provide concrete examples or further explanations. Along the same line, in many places the authors argue complexity without necessarily making clear what these complexities involve and how they relate to the main issues discussed in the article. To give a few examples:

             +On page 9, where Gallist et al. paper is discussed, the authors should try to explain what this “rather complex negotiated practice” involves and why the traditional survey can’t capture some dynamics

             +On page 10, where Dupe et al. paper is discussed, it would be great to get a hint about what these “labour-intensity and complex organisational requirements of these models” are.

 This keeps the barriers discussion a bit abstract. A possible solution is to use some of the  Living Lab cases (or other examples) to explain the issues/complexities. In any case, Living lab cases are very interesting and quite convincing. However, placing them in a separate section at the end dilutes their impact. It would be great to integrate these examples directly into the "Barriers" sections.

 -Last but not least, the distinction between Level 2 (Ecosystems) and Level 3 (SES) is currently not well differentiated.  The data disempowerment discussion under SES could arguably fit in Level 2. Also, the authors may consider extending the Level 3 discussion beyond data disempowerment. For example, the article mentions that women are sidelined in agricultural lobbying groups and tech design, leadership, and decision-making roles. This could be used as a distinct Level 3 barrier related to exclusion from digital governance and institutional leadership.

**Analytical depth, causal mechanisms, policy discussion:**

 -The conclusions are plausible and well aligned with existing policy debates, but the link between barriers and outcomes remains descriptive mainly. The paper would benefit from explicitly articulating causal mechanisms, for example: gender-blind datasets => male-norm software design => lower female adoption => reduced data visibility => continued policy neglect.

**Role of AI and LLM-based tools beyond credit scoring:**

 The discussion of artificial intelligence is currently confined almost exclusively to credit scoring and risk assessment tools. This underplays the potential of AI to address several of the core barriers identified in the paper, particularly *limited access to information*, *digital literacy constraints*, and *language barriers*.

 The authors should briefly discuss how large language model (LLM)-based chatbots could transform access to agricultural advisory services. Unlike traditional digital platforms, LLM-based systems are increasingly multimodal, allowing interaction through voice, images, and text. This is particularly relevant for women farmers who may face literacy constraints or time poverty. Voice-based interaction in local languages could significantly reduce informational asymmetries in extension services, while visual interfaces (e.g., photo-based crop diagnostics) may bypass formal training requirements.

 Moreover, because these tools are typically deployed via cloud infrastructure, they reduce the need for high-end local hardware. This feature is particularly relevant in mobile-only households, a group in which women are overrepresented. As such, LLM-based systems may help alleviate the *inadequate device access* problem identified in the article and enable inclusion without requiring costly capital investments at the household level.

 On the policy side, this suggests that sustainable digitalisation strategies should not only regulate algorithmic bias in financial products, but also actively support the development of public, multilingual, voice-enabled AI advisory tools embedded in extension services. This would strengthen the policy relevance of the framework and connect the conceptual analysis more directly to emerging technological trajectories.

**-**The article would also benefit from briefly contrasting the current *digital* transformation with historical *mechanical* technological changes. For instance, recent research by Ager et al. (2026) demonstrates that the introduction of milking machines in Norway after WWII, a labour-saving mechanical innovation, displaced women from traditional farming roles, ultimately pushing them into higher-skilled urban employment. This historical case highlights a substitution effect where technology replaces manual labour.

 In contrast, the digital technologies discussed in this article (AI, IoT) often function as cognitive layers that influence decision-making and governance rather than purely replacing physical tasks. Unlike the clear-cut displacement seen in Ager et al., digital transformation can either empower women by reducing physical barriers and connecting them to markets, or exclude them through data bias and other barriers. Explicitly discussing this shift would deepen the paper’s theoretical contribution.

**Minor comments:**

 -On page 8, it would be great to make clear which comparative studies of time use are being referenced (if any other than Campana et al paper).

 -Typo on page 10: “In addition , Women’s roles… =>“In addition , women’s roles…”

Is the study design appropriate and does the work have academic merit?

Yes

Is the work clearly and accurately presented and does it cite the current literature?

Yes

If applicable, is the statistical analysis and its interpretation appropriate?

Not applicable

Are all the source data underlying the results available to ensure full reproducibility?

Partly

Are the conclusions drawn adequately supported by the results?

Yes

Are sufficient details of methods and analysis provided to allow replication by others?

Partly

Reviewer Expertise:

Gender and economic growth, corporate governance, financial intermediation

I confirm that I have read this submission and believe that I have an appropriate level of expertise to confirm that it is of an acceptable scientific standard, however I have significant reservations, as outlined above.

## References

[1] : Gender-Biased Technological Change: Milking Machines and the Exodus of Women from Farming. *American Economic Review* .2026;116(1) : 10.1257/aer.20240167 246-286 10.1257/aer.20240167

# Response

**Published:** 2026-03-24

**Authors:** Barbra Comfort Akello

**Affiliations:** Agricultural, Food and Agro-Environmental Sciences, Not Applicable, Pisa, Italy

We thank the reviewer for his insightful and constructive comments, which have improved the quality of this manuscript.

**Reviewer’s comment 1**

*Methodology and conceptual definitions*

 The use of the CODECS framework is appropriate and academically interesting. However, the paper lacks a clearly articulated study design. It is unclear: (1) how literature and policy documents were selected (search strategies or databases used), (2) how many Living Labs were included and on what basis, (3) how evidence was mapped onto the three CODECS levels (coding and categorisation procedures). The study currently reads more like a narrative synthesis than a systematic application of a framework. The authors may consider extending the methodological approach section to address this issue.

**Authors’ response**

 We have expanded the methodological section to better articulate the study design and the systematic application of the CODECS framework. The revisions are presented in the following text (in blue).

*Methodological approach*

 This study employs a qualitative approach and draws on three complementary sources of qualitative evidence.

 Peer-reviewed academic literature was used to establish the conceptual and empirical foundations of the analysis. Relevant studies were identified through targeted searches of major academic databases, such as Google Scholar, Web of Science, and Scopus, and through snowballing citation tracking. Source selection was based on conceptual relevance to gender, agriculture, rural development, and digitalisation, rather than exhaustive coverage. Publications from 2010 onwards were prioritised to reflect developments in digital agriculture, while earlier foundational works were included as needed for theoretical context. Only studies focusing on European countries or comparable developed-country contexts were used to ensure contextual relevance.

 In addition, policy and institutional documents, including EU strategies, parliamentary reports, gender equality assessments, and publications from international organisations, were used. These materials were sourced from official EU repositories, institutional databases, and project websites. Documents were selected for their relevance to agricultural policy, rural development, digital transformation strategies, and gender equality initiatives, with particular attention to those highlighting governance structures, institutional priorities, and policy frameworks influencing digitalisation in agriculture.

 Furthermore, insights were obtained from documented outputs of the CODECS Living Labs, including project reports, technical deliverables, and publicly available summaries. From the full set of 20 Living Labs, two cases were selected based on their explicit engagement with gender-related issues, to offer illustrative insights into how these dynamics may manifest in practice, rather than providing comprehensive case analysis.

 All data were analysed using a deductive coding approach aligned with the three analytical levels of the CODECS framework. Identified barriers and enabling conditions were assigned to levels according to their primary locus of influence, including operational access and decision-making at the farm level (Level 1), participation in platforms, markets, advisory systems, and organisational networks (Level 2), and structural power relations embedded in governance arrangements, data infrastructures, and institutional leadership (Level 3). Cross-level interactions and feedback effects were documented when issues spanned multiple levels.

**Reviewer’s comment 2**

 Related to the second issue mentioned, the article uses Living Lab cases, which are very insightful. Yet it would be great to provide more details, at least for one or two specific examples, describing how women were engaged, which digital tools were adapted or redesigned, and what changed in practice. This would considerably enhance the credibility of the empirical contribution.

**Authors’ response**

 We would like to clarify that in this article, Living Lab cases are not used as primary empirical case studies but rather as illustrative examples to support broader analytical insights derived from the literature and project-based data. Their role is therefore to demonstrate how identified structural challenges and gendered dynamics manifest in practice, rather than to provide in-depth case analysis.

 To improve clarity, we have revised the manuscript by removing the standalone section on Living Labs and integrating the selected cases as illustrative examples within the “barriers” section. An example of how the integration was done is shown below (Blue text).

***Low digital literacy and confidence.*** Digital literacy is an enabling resource in data-driven agricultural systems, yet gendered disparities in skills and confidence continue to limit women’s meaningful engagement with digital technologies. While aggregate indicators suggest a narrowing gender gap in basic digital skills among younger cohorts in the EU, these figures mask persistent, structurally embedded exclusion that disproportionately affects older rural women, particularly those over 45 years working in agriculture ^31^ . This divergence underscores a limitation in current policy approaches, which often prioritise entry-level digital inclusion but overlook the uneven distribution of capabilities necessary for effective and autonomous technology use.

 Literacy gaps stem from cumulative structural inequalities. Lower levels of formal education among older rural women, especially in Southern and Eastern Europe constrain their capacity to acquire digital skills ^32^ . According to the United Nations Educational, Scientific and Cultural Organisation (UNESCO) ^33^, women with only primary education are less likely to use digital tools, while those with secondary education are up to six times more likely to participate in digital environments. However, education alone does not fully account for the persistence of the digital literacy gap. Rural infrastructural deficits, such as limited access to training, inconsistent broadband quality, and demographic pressures including ageing populations and youth outmigration, exacerbate educational shortfalls and contribute to long-term exclusion from digital opportunities. ^33^ .

 Moreover, digital inequality is influenced not only by deficits in technical skills but also by variations in confidence and perceived competence. Socio-cultural norms that associate technology with masculinity undermine women’s self-efficacy, particularly regarding advanced applications such as financial management platforms or farm decision-support systems, leading to an aversion to exploring advanced digital features ^34^ . Findings from the Horizon 2020 DESIRA project show that while rural women frequently use digital tools for routine or small-scale farming tasks, they are more hesitant to adopt advanced technologies. This pattern demonstrates that digital exclusion is stratified rather than absolute, with women more likely to engage in less advanced uses while remaining excluded from domains that provide greater strategic and economic benefits ^35^ . Additionally, digital interfaces that rely on technical language, assume prior expertise, or are available only in dominant national languages create further barriers for users with varying literacy levels or linguistic backgrounds. As a result, women are less likely than men to use digital technologies for fundamental tasks, with estimates indicating a gap of approximately 25% in functional usage ^33^ . The distinction between access and effective use is important, as nominal access to digital tools does not lead to empowerment if users lack the confidence and capability to use them strategically. This limitation constrains the ability to translate digital access into agency and economic outcomes, thereby perpetuating existing inequalities.

 Initiatives such as the Agri-Digital Bildung Living Lab in Germany demonstrate potential strategies for addressing digital literacy gaps by integrating experiential learning, vocational training, and on-farm experimentation. Embedding digital skill development within real production contexts and fostering collaboration among students, farmers, educators, and researchers can help bridge the gap between access and effective use. However, the effectiveness of these initiatives depends on broader structural conditions, including sustained access to training, institutional support, and the redistribution of time and resources necessary for women’s participation ^36^.

**Reviewer’s comment 3**

 Several concepts remain under-specified, for example, *“male farming practices”* in the discussion of design bias in agritech tools. To improve the article's clarity, the authors should provide concrete examples or further explanations.

**Authors’ response**

 We have revised the manuscript to clarify the previously under-specified concept of “male farming practices.” Where appropriate, we have also removed or reconceptualised terms to improve clarity and conceptual precision throughout the manuscript.

**Reviewer’s comment 4**

 Along the same line, in many places the authors argue complexity without necessarily making clear what these complexities involve and how they relate to the main issues discussed in the article. To give a few examples:

 On page 9, where Gallist et al. paper is discussed, the authors should try to explain what this “rather complex negotiated practice” involves and why the traditional survey can’t capture some dynamics

**Authors’ response**

 We have revised the discussion of Gallistl et al. (2021) to clarify the concept of “complex negotiated practice,” both in that section and throughout the manuscript. Specifically, we now elaborate on how digital non-use encompasses practices such as shared device use, dependence on others for digital tasks, and selective or situational engagement with technologies. Additionally, we clarify that traditional survey approaches often fail to capture these dynamics because they rely on binary measures of use versus non-use, thereby overlooking relational, context-dependent, and informal forms of digital engagement. The revisions are indicated in blue text below.

***Inadequate device access and connectivity.*** Policy discourse frequently frames access to digital devices and connectivity as a technical matter of infrastructure provision. However, observed inequalities in connectivity stem not only from coverage gaps but also from unequal ownership, control, and use of digital resources ^37^. Gallistl *et al*. ^38^in a study with older adults in Austria, challenge this reductionist perspective by demonstrating that digital non-use is a negotiated practice shaped by social, cultural, and generational expectations. These practices transcend a simple binary between use and non-use, encompassing nuanced forms of engagement such as relying on others for digital tasks, sharing devices within family or community networks, or selectively engaging with certain technologies while avoiding others. However, current digital interventions typically prioritise connectivity metrics and overlook the lived experiences of exclusion that extend beyond coverage maps and broadband speeds. Consequently, initiatives that focus exclusively on rural broadband expansion risk reinforcing, rather than mitigating digital marginalisation.

 Power dynamics within households and communities further shape access to digital technologies. Perifanou and Economides ^39 ^demonstrate that persistent gender inequalities in rural contexts shape the allocation of devices and digital resources. When technology and digital skills are viewed as scarce, they are often allocated preferentially to men or younger family members. As a result, the prevailing assumption that rural women lack digital skills often reflects structural exclusion rather than individual capability. Addressing this issue requires more than providing training opportunities but also confronting patriarchal norms that dictate who may access, own, or control digital tools. Elena-Bucea *et al*. ^40^similarly contend that the affordability barrier extends beyond the initial cost of devices or internet subscriptions. Ongoing income disparities and precarious rural economies mean that maintaining connectivity involves recurring expenses, which can be especially burdensome for single-income or female-headed households. Policies focused solely on one-time device provision, therefore fail to account for the sustained financial commitments necessary for digital access. Furthermore, the exclusion of rural women from digital resources has broader implications for families and communities. Limited access restricts participation in agricultural innovation, e-government services, and social networks, thereby perpetuating cycles of marginalisation and economic disadvantage. These dynamics underscore the limitations of market-driven digital inclusion strategies that narrowly emphasise infrastructure and consumer access.

 Van Deursen and Van Dijk ^37 ^highlight the limitations of equating internet connectivity with digital empowerment. As basic access becomes more widespread, inequalities increasingly manifest in material differences such as the number, quality, and diversity of devices available, as well as the ongoing costs of maintaining them. In rural Europe, this is evident in the rise of mobile-only households, many of which are led by women. While smartphones enable basic communication, they are often insufficient for more complex digital activities. Tasks like managing agricultural subsidy applications, accessing detailed farming data, or participating in online education typically require larger screens, stable connections, and more advanced devices. As a result, dependence on mobile devices can limit users to passive or limited forms of online engagement. When digital interventions fail to address these material disparities, they risk presenting a superficial appearance of inclusion while deeper forms of exclusion persist.

**Reviewer’s comment 5**

 On page 10, where Dupe et al. paper is discussed, it would be great to get a hint about what these “labour-intensity and complex organisational requirements of these models” are.

 This keeps the barriers discussion a bit abstract. A possible solution is to use some of the Living Lab cases (or other examples) to explain the issues/complexities. In any case, living lab cases are very interesting and quite convincing. However, placing them in a separate section at the end dilutes their impact. It would be great to integrate these examples directly into the "barriers" sections.

**Authors’ response**

 In response to this comment, we have revised the text to clarify the tasks associated with short-supply-chain models. Additionally, we have integrated Living Lab examples directly into the “barriers” section. These revisions are presented below (in blue text).

***Market access constraints.*** Market constraints arise from the disproportionate concentration of women in small-scale or subsistence farming ^20^. Smaller landholdings typically yield lower output volumes, restricting the ability to generate consistent marketable surpluses. Participation in wholesale, export, and other high-value markets generally requires scale, regular supply, and logistical capacity. Consequently, women farmers are frequently restricted to local or low-margin sales channels, which perpetuates income disparities.

 In addition, women’s roles have historically been limited to local markets or household food production, whereas men have dominated the more profitable domains of external trade and large-scale market transactions ^44^. These gendered expectations are reinforced by the unequal distribution of unpaid care work, exemplified in Lithuania, where women predominantly bear the responsibilities of childcare and domestic labour. This dual burden restricts women’s time, mobility, and ability to develop business networks or access distant markets, intensifying barriers to market participation ^45^.

 Women are also underrepresented in producer organisations and cooperatives, which are essential for providing market intelligence, facilitating access to new technologies, enabling collective branding, and strengthening price negotiations ^18^. The absence of collective bargaining power and limited access to market information perpetuate a cycle of low returns and weak negotiating positions. Additionally, gender-based biases among buyers and intermediaries frequently result in women’s products being undervalued, compelling them to accept lower farm-gate prices ^21^.

 While short supply chains, such as farmers’ markets and direct sales can offer women opportunities to bypass traditional barriers and capture higher margins, they also introduce some challenges. According to Dupé *et al*. ^46^, these models require farmers to take on a wide range of responsibilities, including direct marketing, customer communication, order processing, logistics coordination, compliance with certification requirements, financial administration, and participation in collective governance. These tasks increase transaction frequency and demand sustained relational and administrative engagement, often extending beyond standard working hours. For women farmers who disproportionately shoulder unpaid care and household responsibilities, such demands may constitute a structural barrier. The Cloughjordan Living Lab in Ireland exemplifies both the challenges and transformative potential of short supply chains. Its user-owned digital food hub allows producers to upload products to an online marketplace, manage orders, and coordinate deliveries to a shared collection point where payments are centrally processed and redistributed ^36^. While this system reduces intermediaries and enhances local market access, it also requires continuous digital engagement and logistical coordination. Because the platform was co-developed with participating farmers, it aims to streamline administrative tasks and distribute organisational responsibilities collectively, thereby mitigating rather than exacerbating the labour burdens typically associated with short supply chains.

**Reviewer’s comment 6**

 Last but not least, the distinction between Level 2 (Ecosystems) and Level 3 (SES) is currently not well differentiated. The data disempowerment discussion under SES could arguably fit in Level 2. Also, the authors may consider extending the Level 3 discussion beyond data disempowerment. For example, the article mentions that women are sidelined in agricultural lobbying groups and tech design, leadership, and decision-making roles. This could be used as a distinct Level 3 barrier related to exclusion from digital governance and institutional leadership.

**Authors’ response**

 To address this issue, we have revised the theoretical framework to more clearly differentiate the analytical focus of each level. Level 2 is now explicitly defined as capturing meso-level interactions among actors within digital ecosystems, with particular emphasis on coordination, network structures, and access to information flows. In contrast, Level 3 is clarified as referring to the broader institutional and governance context, specifically how rules, policies, and power relations shape digital ecosystems. We have also strengthened the Level 3 analysis. In addition to retaining data disempowerment as a key dimension, we now include the gender-blind design of technologies as a Level 3 consideration and expand the discussion to address women’s exclusion from digital governance and institutional leadership, as indicated in the blue text below.

*Theoretical Framework*

 This study adopts an empowerment-based perspective to analyse how digitalisation reshapes gender relations in EU agriculture. It draws on the framework developed by Kabeer ^8^, which conceptualises empowerment as a process through which individuals gain the capacity to make strategic life choices in contexts where this ability has been systematically constrained. This approach is particularly suited to the analysis of gender inequality in agriculture, as it moves beyond descriptive accounts of participation to examine the underlying power relations that structure access to resources, decision-making, and outcomes.

 Kabeer’s framework distinguishes between three interrelated dimensions of resources, agency, and achievements. Resources encompass not only material assets such as land, capital, and technology, but also human and social resources, including skills, knowledge, and networks. Crucially, access to these resources is mediated by institutional rules and social norms, meaning that their distribution reflects existing power structures rather than neutral allocation mechanisms. Agency refers to the capacity to define goals and act upon them, extending beyond observable decision-making to include processes of negotiation, resistance, and the ability to envision alternative futures. Achievements represent the realised outcomes of these processes, indicating the extent to which individuals are able to translate resources and agency into meaningful improvements in their lives.

 While widely applied in development studies, the framework requires adaptation to capture the specific dynamics of digital transformation in advanced agricultural systems. Kabeer’s model does not explicitly account for the ways in which digital technologies mediate access to resources, reshape forms of agency, and influence institutional structures. Digitalisation introduces new forms of power, including control over data, algorithmic decision-making, and platform-based coordination, which cannot be fully understood through traditional resource-based analyses alone. As such, this study extends the empowerment framework by explicitly incorporating digital infrastructures as sites where power is produced and exercised.

 To operationalise this perspective, the study integrates Kabeer’s framework with the multi-level analytical structure developed in the CODECS project ^17^. This integration addresses a key limitation of empowerment-based approaches, which often focus on individual or household-level dynamics while under-theorising the broader systems within which these processes unfold. The CODECS framework enables the analysis of digitalisation as a system-level transformation, linking micro-level experiences of empowerment to meso and macro-level institutional dynamics.

 The analysis is structured across three interconnected levels.

 Level 1: Digitisation of business processes

 At this level, digitalisation affects the distribution of resources and the exercise of agency through access to technologies, digital skills, and decision-making authority. Gendered disparities at this level may manifest in unequal access to digital tools, limited participation in technology adoption decisions, and differential control over digitally mediated production processes. These dynamics directly influence empowerment outcomes, including productivity, income, and professional recognition.

 Level 2: Digitalisation of ecosystems

 At the ecosystem level, digitalisation shapes interactions among a broader set of actors, including advisory services, cooperatives, platforms, and markets. Here, power operates through network structures, institutional norms, and access to information flows. Participation in digital ecosystems can enable forms of collective agency and social capital accumulation but may also reinforce exclusion if governance structures remain male-dominated or if entry barriers such as digital literacy or platform access are unevenly distributed.

 Level 3: Socio-ecological systems transformation

 At this level, digitalisation is embedded within wider policy frameworks, regulatory regimes, and socio-economic institutions that shape the distribution of resources and opportunities. This includes control over data infrastructures, standard-setting processes, and the design of digital policies. These structures determine whether digital transformation reinforces existing gender inequalities or creates conditions for more transformative change by redistributing power and enabling new forms of participation.

 By linking empowerment processes across these three levels, the framework enables a more comprehensive analysis of how digitalisation reshapes gendered power relations in agriculture.

*Barriers at the level of digital transformation of socio-ecological systems*

 At this level, barriers are embedded in data systems, governance structures, and institutional priorities, shaping who is visible, who participates, and who benefits from digital transformation of agriculture.

***Gender-blind design of technologies.*** The design and implementation of agricultural technologies have historically presumed user neutrality, implicitly treating male experiences, work patterns, and physical characteristics as the normative standard ^52^. This presumption has led to the exclusion and marginalisation of women at multiple levels of agricultural participation. Ergonomic biases are particularly evident in the development of farming equipment, as tools and machinery are frequently designed based on average male body dimensions and strength profiles ^53^. As a result, women often face ergonomic mismatches that render certain tasks more physically demanding or hazardous. These mismatches can reduce efficiency, increase fatigue, elevate injury risks, and reduce adoption of technologies, thereby constraining productivity and perpetuating gendered divisions of labour ^54^.

 Moreover, even when gender issues are acknowledged, insufficient data impedes the development of targeted interventions, training materials, or support services that address women’s specific needs and contexts ^55^^,^^56^. Women’s work, which is disproportionately concentrated in part-time, seasonal, and unpaid labour, is often underrepresented or omitted from official data collection frameworks ^57^. Additionally, self-reporting practices often reflect internalised social norms, with women more likely to identify as helpers rather than primary farmers. These dynamics not only represent descriptive limitations but also actively generate a distorted portrayal of the agricultural workforce, positioning male, full-time farm holders as the implicit reference category ^1^. As a result, women’s contributions are not merely overlooked but are structurally misrecognised within the data systems that inform policy and resource allocation ^58^.

 EU policymaking, including the design and implementation of the Common Agricultural Policy (CAP) depends on statistical indicators to determine eligibility, allocate subsidies, and assess performance. When these indicators are derived from gender-blind or incomplete datasets, they reinforce a narrow model of farming that reflects male-dominated ownership and production structures ^1^, ^5^. Consequently, support schemes, advisory services, and research priorities are not only misaligned with women’s experiences and needs but are also structurally predisposed to exclude them. In this way, gender inequality becomes embedded within the metrics and categories that shape agricultural systems.

***Data disempowerment.*** As farm-level data becomes increasingly central to innovation, decision-making, and market coordination, the governance of data rights becomes critical ^48, 57, 59^. However, existing frameworks around data ownership, consent, and usage fail to account for intra-household power asymmetries and gendered differences in digital literacy and decision-making power ^55, 60^. Women may formally consent to data sharing while lacking control over how that data is used, monetised, or integrated into platform-based systems ^61^. As a result, women remain underrepresented in datasets that shape policy and innovation, while they are simultaneously incorporated into data extraction processes under unequal conditions. As Van der Burg *et al*. ^62^ suggest, such arrangements risk positioning women not as beneficiaries of digital transformation, but as peripheral data subjects within systems that generate value elsewhere.

 Although gender mainstreaming has been formally incorporated into EU policy frameworks, its implementation often remains procedural rather than transformative ^4^. Instruments such as gender impact assessments, training programmes, and gender budgeting are typically added to existing systems without modifying the underlying logic of eligibility, measurement, or accountability ^63^. This approach leads to institutional inertia, where the appearance of inclusion persists alongside structurally biased policy architectures. Furthermore, the lack of robust monitoring mechanisms limits the ability to identify or address these systemic distortions ^1^.

***Exclusion from digital governance and institutional leadership. *** Digitalisation in agriculture constitutes a politically mediated transformation, rather than merely a process of technological adoption ^64^. It is shaped by coalitions of state agencies, producer organisations, corporate actors, and expert communities that collectively define strategic priorities, allocate resources, and design regulatory frameworks ^59^. Within these arenas, women and gender-focused organisations remain underrepresented, concentrating agenda-setting power among actors whose perspectives are often primarily oriented toward productivity and market competitiveness ^65^. This imbalance helps explain why gender equality objectives in EU policy frameworks frequently remain peripheral to dominant priorities such as efficiency, innovation, and trade ^4^.

 Furthermore, the governance of digital agriculture, including data standards, interoperability frameworks, innovation funding, and the regulation of platforms and algorithms, is likewise negotiated in spaces where women are underrepresented and rarely occupy senior decision-making roles ^66^. As a result, implicit assumptions about who qualifies as a farmer, innovator, or legitimate data subject risk becoming embedded in the design of digital systems and policies.

 Similar dynamics are evident at the national level, where ministries, advisory bodies, and industry-led initiatives responsible for digital strategies in agriculture and rural development often reproduce existing gender imbalances. Even where women’s organisations are formally included, their influence may remain limited due to restricted access to key decision-making spaces and insufficient financial or technical resources to engage effectively in complex debates on data governance, artificial intelligence, and platform regulation ^2^.

**Reviewer’s comment 7**

*Analytical depth, causal mechanisms, policy discussion*

 The conclusions are plausible and well aligned with existing policy debates, but the link between barriers and outcomes remains descriptive mainly. The paper would benefit from explicitly articulating causal mechanisms, for example: gender-blind datasets => male-norm software design => lower female adoption => reduced data visibility => continued policy neglect.

**Authors’ response**

 We have clarified these pathways by delineating the mechanisms by which barriers produce downstream effects. An example is illustrated by the blue text below.

***Gender-blind design of technologies.*** The design and implementation of agricultural technologies have historically presumed user neutrality, implicitly treating male experiences, work patterns, and physical characteristics as the normative standard ^52^. This presumption has led to the exclusion and marginalisation of women at multiple levels of agricultural participation. Ergonomic biases are particularly evident in the development of farming equipment, as tools and machinery are frequently designed based on average male body dimensions and strength profiles ^53^. As a result, women often face ergonomic mismatches that render certain tasks more physically demanding or hazardous. These mismatches can reduce efficiency, increase fatigue, elevate injury risks, and reduce adoption of technologies, thereby constraining productivity and perpetuating gendered divisions of labour ^54^.

 Moreover, even when gender issues are acknowledged, insufficient data impedes the development of targeted interventions, training materials, or support services that address women’s specific needs and contexts ^55^^,^^56^. Women’s work, which is disproportionately concentrated in part-time, seasonal, and unpaid labour, is often underrepresented or omitted from official data collection frameworks ^57^. Additionally, self-reporting practices often reflect internalised social norms, with women more likely to identify as helpers rather than primary farmers. These dynamics not only represent descriptive limitations but also actively generate a distorted portrayal of the agricultural workforce, positioning male, full-time farm holders as the implicit reference category ^1^. As a result, women’s contributions are not merely overlooked but are structurally misrecognised within the data systems that inform policy and resource allocation ^58^.

 EU policymaking, including the design and implementation of the Common Agricultural Policy (CAP) depends on statistical indicators to determine eligibility, allocate subsidies, and assess performance. When these indicators are derived from gender-blind or incomplete datasets, they reinforce a narrow model of farming that reflects male-dominated ownership and production structures ^1^, ^5^. Consequently, support schemes, advisory services, and research priorities are not only misaligned with women’s experiences and needs but are also structurally predisposed to exclude them. In this way, gender inequality becomes embedded within the metrics and categories that shape agricultural systems.

**Reviewer’s comment 8**

*Role of AI and LLM-based tools beyond credit scoring*

 The discussion of artificial intelligence is currently confined almost exclusively to credit scoring and risk assessment tools. This underplays the potential of AI to address several of the core barriers identified in the paper, particularly *limited access to information*, *digital literacy constraints*, and *language barriers*.

 The authors should briefly discuss how large language model (LLM)-based chatbots could transform access to agricultural advisory services. Unlike traditional digital platforms, LLM-based systems are increasingly multimodal, allowing interaction through voice, images, and text. This is particularly relevant for women farmers who may face literacy constraints or time poverty. Voice-based interaction in local languages could significantly reduce informational asymmetries in extension services, while visual interfaces (e.g., photo-based crop diagnostics) may bypass formal training requirements.

 Moreover, because these tools are typically deployed via cloud infrastructure, they reduce the need for high-end local hardware. This feature is particularly relevant in mobile-only households, a group in which women are overrepresented. As such, LLM-based systems may help alleviate the *inadequate device access* problem identified in the article and enable inclusion without requiring costly capital investments at the household level.

 On the policy side, this suggests that sustainable digitalisation strategies should not only regulate algorithmic bias in financial products, but also actively support the development of public, multilingual, voice-enabled AI advisory tools embedded in extension services. This would strengthen the policy relevance of the framework and connect the conceptual analysis more directly to emerging technological trajectories.

**Authors’ response**

 We have added a new subsection, “Artificial intelligence and the reconfiguration of gendered barriers,” immediately following the “barriers” section in response to the reviewer’s suggestions. This addition also highlights potential pitfalls of AI, providing a balanced perspective that recognises its transformative potential while cautioning against unintended exclusion. The added section is shown in blue text below.

***Artificial intelligence and the reconfiguration of gendered barriers***

 Recent advances in artificial intelligence, particularly Large Language Models (LLMs), are increasingly positioned as gender-transformative tools capable of reshaping agricultural systems ^67^. Multimodal functionalities incorporating voice, text, and image interaction reduce traditional literacy and language barriers. However, research by Aneja et al. ^68^ highlights that because many LLMs are trained on formal scientific journals and administrative datasets, they often produce outputs laden with formal jargon that fail to recognise the non-standard terms used by women farmers for cropping practices. This linguistic mismatch can reinforce a digital divide even as it attempts to bridge it ^69^.

 Effective engagement with AI necessitates not only digital literacy but also a design-by-inclusion approach grounded in gender equality, diversity, and inclusion principles ^70^. Reviews of AI bias in agriculture highlight that, without such intentional design, AI systems risk perpetuating algorithmic bias, as demonstrated when speech recognition programs or search results favour the demographics most represented in their training data ^71^ As a result, participatory engagement is increasingly recognised as essential for enhancing the relevance and usability of AI tools among marginalised groups ^70^.

 Additionally, although AI-enabled automation has the potential to alleviate time poverty by streamlining administrative and labour-intensive tasks, its effectiveness is significantly influenced by prevailing household norms. A study by Di and Li ^72^ indicates that digital tools could theoretically reduce housework by approximately 30%; however, urban-rural disparities in adoption restrict these benefits for rural women. Moreover, the time saved through mechanisation or automation is often reallocated to additional unpaid care, domestic chores, or child-rearing activities, rather than being redirected toward income-generating or decision-making roles ^73^.

 At the decision-making level, AI-based advisory tools offer private, on-demand information that can circumvent male-dominated extension networks. Reeves *et al*. ^69^ report that machine-supported decision-making increases female participation in agricultural training by optimising for gender-specific preferences, such as session timing and location. However, intra-household hierarchies continue to determine ownership of digital credentials and devices ^64^ . In predominantly patriarchal heritage contexts, men frequently control access to technology or interpret AI-generated guidance, so formal access does not necessarily translate into decision-making autonomy.

 AI technologies also shape epistemic authority by prioritising algorithmically generated metrics over experiential and relational knowledge. When AI systems are trained on formal, biased datasets, they risk marginalising the indigenous and context-specific expertise of women who have historically been excluded from formal agricultural institutions ^74^ . Prioritising AI-derived outputs may further diminish recognition of women's expertise unless gender-responsive data practices and participatory design are integrated from the outset ^68^. Excluding diverse knowledge forms not only limits system relevance but also reinforces cultural hierarchies that marginalise women’s contributions to agricultural innovation ^74^ .

 AI's dependence on large-scale datasets requires robust, gender-responsive data governance to prevent structural disempowerment. When datasets are gender-blind or reflect historical biases, they institutionalise inequality by rendering women invisible as economic actors while exposing them as data subjects ^71^. Addressing these feedback loops necessitates frameworks that prioritise data rights, transparency, and the deliberate inclusion of women's labour and economic contributions in AI training processes to ensure that digital transformation does not exclude marginalised farmers ^53^.

**Reviewer’s comment 9**

 The article would also benefit from briefly contrasting the current *digital* transformation with historical *mechanical* technological changes. For instance, recent research by Ager et al. (2026) demonstrates that the introduction of milking machines in Norway after WWII, a labour-saving mechanical innovation, displaced women from traditional farming roles, ultimately pushing them into higher-skilled urban employment. This historical case highlights a substitution effect where technology replaces manual labour.

 In contrast, the digital technologies discussed in this article (AI, IoT) often function as cognitive layers that influence decision-making and governance rather than purely replacing physical tasks. Unlike the clear-cut displacement seen in Ager et al., digital transformation can either empower women by reducing physical barriers and connecting them to markets or exclude them through data bias and other barriers. Explicitly discussing this shift would deepen the paper’s theoretical contribution.

**Authors’ response**

 We have included a brief comparison of historical mechanical technological changes and contemporary digital transformation in the “Introduction” section (blue text), as shown below.

*Introduction*

 Agriculture in the European Union (EU) remains characterised by deep-rooted gender inequalities, despite decades of political efforts to achieve equality ^1^ . Women continue to face significant barriers in accessing land, finance, education, technology, as well as meaningful decision-making power, leaving them at a disadvantage compared to men in almost all areas of agriculture and rural life ^2^ . Although gender equality is frequently cited in rural development objectives, many initiatives fail to address the underlying socio-cultural structures that reinforce this marginalisation ^3^ . As Shortall and Marangudakis ^4^ highlight, a fundamental structural imbalance of influence underpins the ineffectiveness of these measures. Dominant agricultural lobbying groups wield substantial influence over EU policy priorities, while gender-focused advocacy remains comparatively under-resourced and lacks robust legal and institutional backing. As a result, gender concerns are systematically sidelined in EU’s agricultural policy framework.

 The outcome of this neglect is adverse for women in rural areas, contributing to lower productivity, fewer entrepreneurial opportunities, limited economic independence, and diminished quality of life ^1^^, ^^4^. This marginalisation is further evidenced by the reality that although women make up 35% of the agricultural labour force in the EU and contribute 31% of total working hours to the economy, their work continues to be undervalued, and their specific needs often remain unaddressed in policy and practice ^5^ . The persistence of these gaps contrasts with the EU’s long-standing commitment to gender equality, enshrined in its treaties and reinforced by milestones such as the 1957 Treaty of Rome, the 1996 communication on gender mainstreaming, and the Gender Equality Strategy 2020–2025 ^6^ .

 From a gender and development perspective, this problem is best understood through the lens of empowerment, rather than representation or resource allocation alone ^7^. Empowerment is conceptualised here as a process through which individuals gain the ability to make strategic life choices in contexts where such ability has been systematically constrained ^8^. This process unfolds across interconnected dimensions, including access to resources, the exercise of agency, and the institutional conditions that enable or restrict the translation of resources and agency into sustained outcomes. Persistent gender inequalities in agriculture therefore reflect not only disparities in assets or opportunities, but enduring limitations on women’s capacity to influence decisions affecting their labour, livelihoods, and futures ^9^.

 Digitalisation in agriculture is widely promoted in policy and industry circles as a strategic opportunity to redress the structural imbalances ^10^. Narratives associated with this so-called fourth agricultural revolution portray digital technologies such as artificial intelligence (AI), blockchain, and the Internet of Things (IoT) as neutral tools that can reduce labour burdens, improve access to information and markets, and enhance productivity ^11-13^. However, such techno-optimist accounts risk obscuring the social and institutional conditions under which digitalisation unfolds. Digital technologies do not operate in a vacuum as they are embedded in existing socio-technical systems and shaped by prevailing norms, power relations, and governance arrangements ^11^. Consequently, while digitalisation may expand women’s resources and agency, it may also reproduce or intensify gender inequalities by reinforcing existing patterns of exclusion in technologically mediated forms.

 Moreover, contemporary digital transformation differs fundamentally from earlier waves of technological change in agriculture, with distinct implications for gender relations. Historical mechanical innovations were largely characterised by a substitution effect, whereby machines replaced manual labour and directly reshaped rural labour markets. The post-war introduction of milking machines in Norway illustrates this dynamic clearly. Between 1930 and 1970, young rural women displaced from dairy farming were more likely to transition into better-paid, skilled employment, particularly in the public sector and to invest more in higher education ^14^. Although men were also affected by mechanisation, their displacement was less pronounced, and they did not experience comparable occupational upgrading. This pattern reflects a gender-biased technological change that contributed to the masculinisation of Norwegian agriculture, while, paradoxically narrowing long-term gender gaps in employment and education ^14^.

 The digital technologies shaping present day European agriculture, such as AI, data platforms, and IoT, function less as direct substitutes for labour and more as cognitive and institutional layers that mediate information flows, decision-making processes, and governance arrangements ^12^. Rather than simply displacing work, digital systems influence who has access to data, whose knowledge is recognised, and who exercises authority within increasingly data-driven agricultural systems. As a result, digitalisation can generate divergent gendered outcomes. On the one hand, it may empower women by lowering physical barriers, expanding access to markets and knowledge, and enabling new forms of agency, including opportunities for innovation and change agency ^6^. On the other hand, it may reinforce exclusion through biased data infrastructures, unequal digital skills, and male-dominated governance structures, thereby intensifying a triple divide based on gender, digital access, and rural location ^11^. Recognising this shift from labour substitution to cognitive mediation is essential for understanding why digital transformation in agriculture does not produce uniform gender effects.

 Current discussions on digital agriculture in the EU reflect this ambivalence ^10, 15, 16^. While a growing body of literature documents the economic and environmental benefits of digital technologies, far fewer studies systematically evaluate their implications for gender equality ^2^. Where gender is addressed, analyses often remain fragmented, focusing on isolated dimensions such as access to technology, labour participation, or entrepreneurship ^2, 4^. This has resulted in a lack of integrated explanations capable of clarifying how and under what conditions digitalisation of agriculture contributes to women’s empowerment, rather than merely describing its effects ^11^. The absence of a coherent framework capable of linking digital transformation to power, agency, and institutional change limits the analytical and policy relevance of the existing evidence.

 This study addresses this gap by employing an empowerment lens, operationalised through the framework developed in the Horizon project *Maximising the CO-benefits of agricultural Digitalisation through conducive digital ECoSystems* (CODECS). Instead of treating digitalisation as an isolated technological intervention, the analysis examines how empowerment processes develop within agricultural systems experiencing digital transformation. The CODECS framework conceptualises these systems across three interconnected levels: farm business processes, digital ecosystems, and broader socio-ecological systems ^17^. This multi-level perspective enables the identification of how opportunities for empowerment are facilitated or restricted at various scales, and how digital transformation interacts with existing governance structures, norms, and power relations in European agriculture.

**Reviewer’s comment 10**

 On page 8, it would be great to make clear which comparative studies of time use are being referenced (if any other than Campana et al paper).

**Authors’ response**

 We have revised this section to capture comparative studies highlighting gender gaps in time allocation across developed countries, with variations by age, life stage, and welfare regime. Below, we show how we incorporated it in the “barriers” section (Blue text)

***Time and labour burden.*** Women comprise approximately 35% of the agricultural workforce, yet they are often employed in unpaid, part-time, or family-worker roles that are not captured by official statistics ^6^. Domestic and care responsibilities assigned to women reduce the time available for paid employment, skills development, training participation, and decision-making involvement. This results in time poverty, which constrains women's agency ^23^. Over time, these constraints lead to diminished income security, restricted access to social protection, and lower pension accumulation, thereby reinforcing long-term economic vulnerability.

 At the policy level, these inequalities are perpetuated by the sectoral framing of agriculture. As Shortall and Marangudakis ^24 ^argue that EU agricultural policy primarily defines agriculture in terms of land, production, and subsidy distribution, rather than as an occupation shaped by gendered divisions of work. As a result, instruments such as the Common Agricultural Policy (CAP) allocate support based on land ownership and farm size, criteria that systematically favour men, who are more likely to control larger and more profitable holdings. In contrast, women are disproportionately represented in smaller-scale or subsistence-oriented farms, many of which do not qualify for substantial support. This structural disconnect between labour contribution and resource allocation reinforces gendered inequalities in income and investment capacity.

 Gendered expectations influence both the types of tasks women perform and the allocation of time within households. Comparative time-use studies across Europe indicate that countries with more traditional gender norms display greater disparities in both paid and unpaid work. In contrast, more egalitarian welfare regimes and family policies are associated with narrower gaps, suggesting that labour inequalities are equally shaped by institutional context rather than being culturally fixed ^25^.

 Cross-national evidence supports this structural perspective. For example, Román and Gracia ^26^ demonstrate that gender gaps in unpaid care work and paid employment follow a consistent life-course trajectory, widening during adolescence, peaking in early adulthood, and narrowing later in life. The magnitude of these gaps, however, varies considerably across countries, with more pronounced inequalities in Southern Europe compared to more egalitarian welfare contexts such as Sweden. Similarly, Anxo et al. ^27^ show that variations in family policy regimes directly influence the distribution of paid and unpaid labour, highlighting the significance of institutional design in either perpetuating or mitigating gendered time inequalities.

 Nevertheless, increased female labour force participation has not necessarily resulted in economic empowerment. Instead, it is often associated with the growth of part-time, precarious, and low-paid employment. Filandri and Struffolino ^28 ^demonstrate that high female employment rates do not inherently reduce poverty when job quality is poor and social protection is inadequate. In these contexts, labour market inclusion may coexist with, or even exacerbate, economic insecurity. This challenges policy assumptions that participation alone is sufficient for empowerment and underscores the importance of how labour is structured, valued, and compensated.

**Reviewer’s comment 11**

 Typo on page 10: “In addition, Women’s roles… => “In addition, women’s roles…”

**Authors’ response**

 We have corrected this typo as shown below (Blue text)

 In addition, women’s roles have historically been limited to local markets or household food production, whereas men have dominated the more profitable domains of external trade and large-scale market transactions ^44^. These gendered expectations are reinforced by the unequal distribution of unpaid care work, exemplified in Lithuania, where women predominantly bear the responsibilities of childcare and domestic labour. This dual burden restricts women’s time, mobility, and ability to develop business networks or access distant markets, intensifying barriers to market participation ^45^.
